# Bictegravir/emtricitabine/tenofovir alafenamide for HIV: a meta-analysis of efficacy across treatment-naïve and experienced populations and comparative effectiveness versus dolutegravir/lamivudine

**DOI:** 10.3389/fphar.2026.1935865

**Published:** 2026-10-30

**Authors:** Mingxia Cheng, Aiwen Wang, Qingshuang Zhu, Hua Duan, Kangle Guo, Li Li, Shixun Ma, Jin He, Kehu Yang

**Affiliations:** 1 The First Clinical School of Gansu University of Chinese Medicine, Lanzhou, China; 2 Institute of Evidence-Based Medicine, Gansu Provincial Hospital, Lanzhou, China; 3 Department of HIV/AIDS and other Sexually Transmitted Diseases Control and Prevention, Heze Center for Disease Control and Prevention (Heze Health Supervision Institute), Heze, Shandong, China; 4 Department of Scientific Research, Gansu Provincial Hospital, Lanzhou, China; 5 The First Clinical Medical College, Lanzhou University, Lanzhou, China; 6 Evidence-Based Medicine Center, School of Basic Medical Sciences, Lanzhou University, Lanzhou, China

**Keywords:** bictegravir/emtricitabine/tenofovir alafenamide, HIV, meta-analysis, treatment discontinuation, virologic suppression

## Abstract

**Objectives:**

Bictegravir/emtricitabine/tenofovir alafenamide (BIC/FTC/TAF) is a preferred single-tablet regimen for HIV treatment, but head-to-head meta-analytic comparisons between treatment-experienced (TE) and treatment-naïve (TN) individuals are lacking. Additionally, the comparative effectiveness of BIC/FTC/TAF versus dolutegravir/lamivudine (DTG/3TC) remains to be systematically synthesized. We aimed to systematically compare the efficacy of BIC/FTC/TAF between TE and TN people with HIV, and evaluate the comparative effectiveness of BIC/FTC/TAF versus DTG/3TC.

**Methods:**

We searched PubMed, Cochrane Library, Web of Science, and Embase up to September 2026. Observational studies and randomized controlled trials (RCTs) reporting virologic suppression or treatment discontinuation rates in both TE and TN populations receiving BIC/FTC/TAF, or BIC/FTC/TAF versus DTG/3TC were included. Pooled odds ratios (ORs) with 95% confidence intervals (CIs) were calculated. Single-group proportion meta-analyses used a random-effects model, whereas comparative meta-analyses used the prespecified fixed-effect model.

**Results:**

Thirty-one studies were included. For TE participants, the pooled virologic suppression rate was 95% (95% CI: 92%–97%) at 12 months, and for TN participants, the rate was 90% (95% CI: 85%–94%). In design-stratified comparative analyses between BIC/FTC/TAF and DTG/3TC, no statistically significant difference was detected in RCT (OR = 1.46, 95% CI: 0.80–2.66) or cohort studies (OR = 0.98, 95% CI: 0.70–1.39) among TN populations, nor in TE populations (RCT: OR = 1.04, 95% CI: 0.34–3.16; cohort: OR = 0.92, 95% CI: 0.42–2.02). No statistically significant differences were detected for treatment discontinuation (all-cause: OR = 1.02, 95% CI: 0.70–1.49; adverse events: OR = 1.11, 95% CI: 0.55–2.22).

**Conclusion:**

BIC/FTC/TAF achieves high virologic suppression rates and low discontinuation rates in both TE and TN populations, supporting its use as a first-line and switch option. BIC/FTC/TAF and DTG/3TC showed no statistically significant differences in virologic suppression or treatment discontinuation, suggesting both are viable guideline-preferred options. The choice between them should be individualized based on patient characteristics, including prior treatment history, adherence potential, comorbidity profile, and metabolic risk considerations.

## Introduction

Human immunodeficiency virus (HIV) infection remains a major global public health challenge. According to the 2024 global estimates by World Health Organization (WHO) and UNAIDS, an estimated 40.8 million people were living with HIV worldwide at the end of 2024 (WHO, 2026). In 2024 alone, 1.3 million people acquired HIV, and 630,000 people died from HIV-related causes globally. Despite substantial progress in scaling up antiretroviral therapy (ART), with 31.6 million people accessing ART by 2024, HIV continues to drive substantial morbidity, mortality, and health disparities across regions and key populations (UNAIDS, 2026). The epidemic imposes persistent burdens on healthcare systems and underscores the continued need for effective, well-tolerated, and widely implementable first-line and switch regimens in both treatment-naïve (TN) and treatment-experienced (TE) individuals living with HIV.

ART has transformed HIV infection from a fatal disease into a manageable chronic condition, serving as the cornerstone of global HIV response. As of the end of 2024, 77% of people living with HIV (PLWH) globally had access to ART, with 73% achieving sustained viral suppression, a milestone that has drastically reduced AIDS-related mortality and transmission risk (WHO, 2026). The WHO now strongly recommends universal, rapid ART initiation (ideally within 7 days of diagnosis, regardless of CD4^+^ T-cell count) for all PLWH, with the goal of achieving and maintaining viral suppression to improve individual health outcomes and eliminate HIV transmission (WHO, 2025). To meet these goals, modern ART regimens must balance high efficacy, excellent tolerability, minimal drug-drug interactions, and convenient dosing to support long-term adherence. Among the most widely adopted first-line and switch regimens globally, the fixed-dose combination of bictegravir/emtricitabine/tenofovir alafenamide (BIC/FTC/TAF) has emerged as a preferred option in multiple international guidelines, including the 2025 European AIDS Clinical Society (EACS) (Ambrosioni et al., 2026) and US Department of Health and Human Services (DHHS) guidelines (DHHS, 2025). This single-tablet regimen (STR) combines a second-generation integrase strand transfer inhibitor (INSTI) with two nucleoside reverse transcriptase inhibitors (NRTIs), delivering a high genetic barrier to resistance, rapid viral suppression, and favorable renal and bone safety profiles compared to older tenofovir disoproxil fumarate (TDF)-based regimens (Kityo et al., 2025; Schuettfort et al., 2026). Real-world evidence has further confirmed its effectiveness across diverse populations, including TN, TE, and key vulnerable groups, solidifying its central role in contemporary HIV care (Curteis et al., 2025). In addition to BIC/FTC/TAF, the two-drug regimen dolutegravir/lamivudine (DTG/3TC) has also gained widespread acceptance as a guideline-preferred option for both first-line and switch therapy, offering comparable efficacy with reduced cumulative drug exposure and potentially fewer long-term toxicities (Grau et al., 2023; Evitt et al., 2023). Given that both BIC/FTC/TAF and DTG/3TC are now among the most commonly prescribed regimens in clinical practice, accumulating real-world studies have compared their effectiveness and tolerability head-to-head. However, the evidence base remains fragmented, and a meta-analytic synthesis comparing these two regimens has not yet been performed to our knowledge.

Although several meta-analyses have assessed the efficacy of BIC/FTC/TAF in TN and TE populations individually (Chivite et al., 2024; Rashid et al., 2025; Chen et al., 2021), a direct head-to-head meta-analytic comparison of virologic outcomes between these two groups is lacking. Furthermore, although a recent network meta-analysis (Curteis et al., 2025) evaluated the comparative safety of BIC/FTC/TAF versus other antiretroviral regimens in TN people with HIV-1, including DTG-containing regimens, it focused exclusively on safety outcomes in the TN population and did not specifically compare BIC/FTC/TAF with DTG/3TC for virologic suppression or treatment discontinuation, nor did it include TE individuals. A dedicated meta-analytic synthesis comparing the efficacy and discontinuation outcomes of BIC/FTC/TAF versus DTG/3TC across both TN and TE populations is therefore still lacking. Given the differing clinical backgrounds and treatment objectives of TN and TE individuals, as well as the need to clarify the comparative standing of BIC/FTC/TAF relative to DTG/3TC, additional quantitative evidence is needed to guide clinical decision-making. This study therefore aimed to systematically compare the efficacy of BIC/FTC/TAF between TE and TN people with HIV, and evaluate the comparative effectiveness of BIC/FTC/TAF versus DTG/3TC.

## Methods

This study employed a methodical review and meta-analysis approach, following the established guidelines outlined in the Preferred Reporting Items for Systematic reviews and Meta-Analyses (PRISMA). The research protocol was registered with the International Prospective Register of Systematic Reviews (PROSPERO, registry ID: CRD420261376911).

### Search strategy

We searched PubMed, the Cochrane Library, Web of Science, and Embase for studies published from the date of their inception to 1 September 2026. The search was restricted to studies published in English. The main search strategies were as follows: (bictegravir/emtricitabine/tenofovir alafenamide OR B/F/TAF OR BIC/FTC/TAF) AND (human immunodeficiency virus OR HIV OR People living with HIV OR PLWH). To ensure comprehensive coverage, we also searched clinical trial registries, including ClinicalTrials.gov and the WHO International Clinical Trials Registry Platform (ICTRP), and manually screened the reference lists of included studies and relevant reviews to identify additional eligible studies. Complete database-specific search strategies, interfaces, and the exact number of records retrieved from each source are provided in Supplementary Table S1.

### Inclusion and exclusion criteria

This meta-analysis addressed two distinct research questions. The first question compared virologic suppression and treatment discontinuation between TE and TN individuals receiving BIC/FTC/TAF. The second question compared the same outcomes between BIC/FTC/TAF and DTG/3TC, regardless of prior treatment history. For Question 1 (TE vs. TN within BIC/FTC/TAF), studies were eligible if they: (1) interventional trials (such as randomized controlled trials) or observational studies (such as cohort studies) were eligible; (2) enrolled both TE and TN participants receiving BIC/FTC/TAF; (3) reported the number of participants achieving HIV-RNA <50 copies/mL or treatment discontinuation separately for each group. Studies that included only TE or only TN participants were excluded from this analysis. For Question 2 (BIC/FTC/TAF vs. DTG/3TC), studies were eligible if they: (1) directly compared BIC/FTC/TAF with DTG/3TC in any HIV-positive population (TN, TE, or mixed); (2) reported virologic suppression or treatment discontinuation for each regimen. No restriction was placed on prior treatment history, and single-arm studies without a DTG/3TC comparator were excluded from this analysis. Studies with unavailable outcome data, reviews, or animal studies were excluded from both questions.

### Literature selection and data extraction

Two authors independently screened the identified studies for eligibility. Potentially relevant studies were initially identified by title and abstract screening, and those meeting the inclusion criteria were further assessed by full-text reading to determine final inclusion. All studies were screened for potential overlap by comparing author lists, institutional affiliations, enrollment periods, and sample sizes; when uncertainty existed, we contacted the corresponding authors for clarification. Any discrepancies were resolved by consensus.

Core data were extracted independently by the same two authors using a pre-specified standardized form. For each question, full-text articles were assessed using the corresponding eligibility criteria. Studies that met criteria for Question one were extracted for TE vs. TN comparison; studies that met criteria for Question two were extracted for BIC/FTC/TAF vs. DTG/3TC comparison. The extracted information included: (1) basic study characteristics, such as the first author, study design, year of publication, and country/region; (2) participant characteristics, including sample size, distribution of TE and TN individuals, age, and gender; (3) treatment duration; (4) outcome measures along with the corresponding event counts and sample sizes. For observational studies, we preferentially extracted adjusted effect estimates, or propensity score–matched/adjusted estimates) when these were reported in the original studies, in order to minimize confounding. Where adjusted estimates were not reported, we extracted unadjusted event counts and calculated unadjusted odds ratios; and (5) information related to the risk of bias assessment.

### Risk of bias and quality of evidence assessment

The quality of the included cohort studies was evaluated using the Newcastle-Ottawa Scale (NOS) (Wells et al., 2021). This tool assesses three core dimensions: selection of the study groups, comparability of the cohorts, and assessment of the outcome and follow-up. The NOS assigns a maximum total score of nine points, with higher scores indicating better methodological quality and lower risk of bias. For randomized controlled trials (RCTs), the risk of bias was assessed independently using the revised Cochrane risk-of-bias tool for randomized trials (RoB 2.0) (Sterne et al., 2019). The assessment covered five domains: (1) bias arising from the randomization process, (2) bias due to deviations from intended interventions, (3) bias due to missing outcome data, (4) bias in measurement of the outcome, and (5) bias in selection of the reported result. Each domain was judged as “low risk,” “some concerns,” or “high risk,” and an overall risk-of-bias judgment was subsequently derived for each trial. Disagreements between reviewers were resolved through consensus discussion.

We assessed the certainty of evidence for key comparative outcomes using the Grading of Recommendations, Assessment, Development and Evaluations (GRADE) framework (Guyatt et al., 2025a). The certainty of evidence was evaluated across the following domains: risk of bias, inconsistency, indirectness, imprecision, and publication bias. For RCTs, the initial evidence certainty was rated as high, which could be downgraded by one or two levels if serious or very serious limitations were identified in any of the five domains. For observational studies (cohort studies), the initial evidence certainty was rated as low, which could be downgraded further if serious limitations existed, or upgraded if there was a large magnitude of effect, a dose–response gradient, or if plausible confounding would reduce a demonstrated effect or suggest a spurious effect when no effect was observed. The final certainty of evidence was categorized into four levels: high (further research is very unlikely to change confidence in the estimate of effect), moderate (further research is likely to have an important impact on confidence in the estimate and may change the estimate), low (further research is very likely to have an important impact on confidence in the estimate and is likely to change the estimate), and very low (any estimate of effect is very uncertain) (Guyatt et al., 2025b; Guyatt et al., 2025c; Guyatt et al., 2025d).

### Data analysis

All statistical analyses were performed using RevMan software, Stata and the R programming language. For question 1, a meta-analysis of event rates was first conducted based on the single-group pooling method, using the number of events and total sample size as input data, we applied the Freeman-Tukey double arcsine transformation to stabilize variances. Because between-study heterogeneity in event rates was expected and was observed to be substantial, a random-effects model was used for these single-group proportion meta-analyses, as stated in the study protocol. In addition, for Question 1, if potentially overlapping BICSTaR-related studies were identified, only the primary study was included in the meta-analysis, while overlapping sub-studies were presented descriptively and not included in the pooled analysis.The pooled effect sizes were presented as point estimates with corresponding 95% confidence intervals (CI). Statistical heterogeneity was quantified using the I^2^ statistic. For the comparison between TE and TN participants receiving BIC/FTC/TAF, we carefully assessed the comparability of the study populations. Given the substantial baseline differences between TE and TN populations (e.g., TE participants were predominantly virologically suppressed at baseline while TN participants initiated therapy with detectable viremia), we did not perform a direct meta-analytic comparison for the virologic suppression outcome; instead, we presented the findings descriptively. For the discontinuation outcome, we performed meta-analyses between TE and TN only when the reasons for discontinuation and follow-up durations were sufficiently homogeneous across studies. In addition, for Question 1, if potentially overlapping BICSTaR-related studies were identified, only the primary study was included in the meta-analysis, while overlapping sub-studies were presented descriptively and not included in the pooled analysis. For the comparison between BIC/FTC/TAF and DTG/3TC (Question 2), we first evaluated the comparability of the two treatment groups within each study. Where a direct head-to-head comparison was feasible, we conducted meta-analyses and presented the pooled effect sizes as odds ratios (ORs) with 95% CI. Where direct comparison was not feasible due to heterogeneity in study populations, outcome definitions, or severe channeling bias, we summarized the findings descriptively. The choice of effect model was guided by the degree of heterogeneity. We prespecified the fixed-effect model as the default approach in comparative meta-analyses (Question 1: TE vs. TN discontinuation; Question 2: BIC/FTC/TAF vs. DTG/3TC), as only studies with comparable populations, study designs, and follow-up durations were included in each meta-analysis after rigorous *a priori* assessment of clinical and methodological homogeneity. Studies with zero events in both arms were excluded from comparative meta-analyses as they provide no information on the relative effect. The follow-up time points (6 months, 12 months, and ≥24 months) were prespecified based on the most commonly reported time points in HIV clinical trials and real-world studies, with 12 months defined as the primary time point for comparative analyses. To evaluate the robustness of the pooled estimates, sensitivity analyses based on fixed-effect model were performed, this leave-one-out approach was applied to all comparative meta-analyses, including the comparison between TE and TN individuals for treatment discontinuation, as well as the comparison between BIC/FTC/TAF and DTG/3TC for the two outcomes. Heterogeneity was assessed using the I^2^ statistic and Cochran’s Q test; I^2^ values of 25%, 50%, and 75% were considered low, moderate, and substantial, respectively.

## Results

### Literature screening process and results

The study selection process is illustrated in the PRISMA flow diagram (Figure 1). A total of 2412 records were initially identified. After removing 1572 duplicate records, 840 unique records remained for title and abstract screening. Of these, 785 records were excluded based on irrelevance to the research topic, leaving 55 full-text articles to be assessed for eligibility. Ultimately, 31 studies (Yang et al., 2026a; Kim et al., 2026; Yo et al., 2025; Trizzino et al., 2025; Ma et al., 2025; Kim et al., 2025; Inciarte et al., 2025; Hocqueloux et al., 2025; Cecchini et al., 2025; Torralba et al., 2024; d'Arminio Monforte et al., 2024; Nasreddine et al., 2023; Mendoza et al., 2023; Kong et al., 2023; Ambrosioni et al., 2022; Yang et al., 2026b; Wu et al., 2026; Sun et al., 2026; Serrano-Villar et al., 2026; Perez-Valero et al., 2026; Marcelin et al., 2026; Guo et al., 2026; Fan et al., 2026; Ciccullo et al., 2026; Pierone et al., 2025; Liu et al., 2025; Lee et al., 2024; Gan et al., 2024; Knobel et al., 2023; Martin-Torres et al., 2026; Ghosn et al., 2026) were included in the final meta-analysis.

**FIGURE 1:**
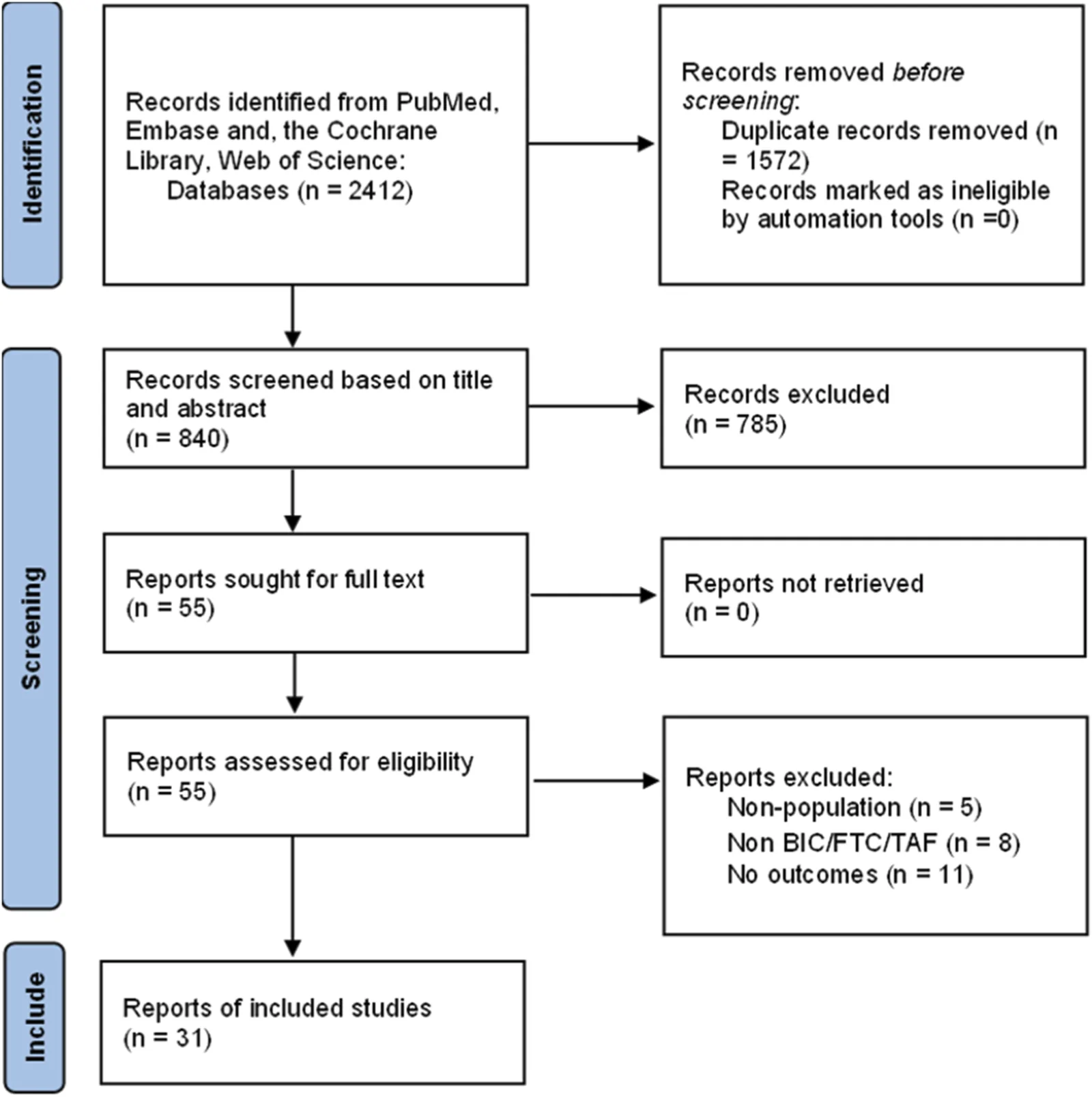
The flowchart of literature screening.

### Description of the included studies

Of the 31 included studies, 15 provided data for the TE-vs-TN comparison within BIC/FTC/TAF (Question 1), and 17 provided direct comparative data between BIC/FTC/TAF and DTG/3TC (Question 2), one study contributed to both analyses. The specific details of the included studies (28 cohort studies and three RCTs) are presented in Table 1. The studies were conducted across multiple regions, including Korea, China, Japan, several European countries, Argentina, and overall Asian populations, with study periods ranging from 2016 to 2025. The total combined sample size for all 31 studies was 41,740 participants, consisting of 33,856 TE and 7.884 TN individuals. Most participants were male, and the mean or median age ranged from approximately 31–61 years across studies. Treatment duration varied across studies, including 6 months, 12 months, 24 weeks, 48 weeks, and up to 69.8 weeks (equivalent to approximately 16 months). The primary reported outcomes were virologic suppression (HIV-RNA < 50 copies/mL) and treatment discontinuation. For the three RCTs, risk of bias was assessed using the RoB 2.0 tool (Supplementary Appendix Table 2). The MIND trial (Perez-Valero et al., 2026) was rated as overall low risk of bias, owing to its double-blind, placebo-controlled design, centralized randomization, and use of an independent adjudication committee for neuropsychiatric adverse events. The INSTINCT trial (Serrano-Villar et al., 2026) and the VOGUE trial (Ghosn et al., 2026) were both rated as overall some concerns, primarily due to their open-label designs, which may have introduced performance and detection bias for subjective outcomes such as patient-reported symptoms and adverse event reporting. However, the primary endpoints of these two trials (inflammatory biomarkers and HIV-1 RNA < 50 copies/mL, respectively) were objectively measured and therefore less susceptible to such bias. The RoB two judgments were made specifically for the outcomes included in our meta-analysis (virologic suppression and treatment discontinuation), not for the original trial primary endpoints. The results of risk of bias assessment showed that among the 28 included cohort studies, the overall quality scores ranged from seven to nine points, with most studies scoring 8 and 9 points. Domain-level assessments across the included cohort studies indicated generally low risk of bias, although the interpretation of total scores should consider the specific domains in which limitations were identified (see Supplementary Appendix Table 3 for details).

**TABLE 1:** The basic characteristics of the included studies.

Study ID	Study design	Location	Period	Question	n (TE/TN)	Sex (Male/Female)	Age	Duration	Outcomes
(Yang et al., 2026a)	Prospective cohort	Asian	2020–2024	PICO1	334 (268/66)	313/21	TN: 31.5 (28.0, 36.0); TE: 42.0 (35.0, 49.0)	48 months	①②
(Kim et al., 2026)	Prospective cohort	Korea	2022-2023	PICO1	1,157 (1033/124)	1,080/77	ALL: 43.3 ± 13.7	48 weeks	①
(Yo et al., 2025)	Prospective cohort	Japan	2020–2023	PICO1	200 (84/116)	198/2	TN: 34.0 (28.0, 44.5); TE: 45.0 (39.0, 50.0)	12 months	①②
(Trizzino et al., 2025)	Prospective cohort	Italy	2019–2025	PICO1	214 (177/37)	160/54	ALL: 60.6 ± 7.3	12 months, 24 months	①
(Ma et al., 2025)	Prospective cohort	China	2021–2024	PICO1	292 (143/149)	197/95	TN: 61.0 (56.0, 68.0); TE: 61.0 (56.0, 68.0)	48 weeks	①②
(Kim et al., 2025)	Ambispective cohort	Korea	2021–2024	PICO1	88 (52/36)	81/7	Median: TN: 32.0; TE: 52.5	24 weeks	①②
(Inciarte et al., 2025)	Prospective cohort	Spain	2019–2021	PICO1	249 (187/62)	205/44	TN: 32.5 (26.0, 39.0); TE: 47.0 (37.0, 55.0)	12 months	①②
(Hocqueloux et al., 2025)	Prospective cohort	France	2019-2019	PICO1	240 (184/56)	190/50	ALL: 50 (40, 58)	12 months	①
(Cecchini et al., 2025)	Prospective cohort	Argentina	2019–2021	PICO1	2,671 (2,356/315)	1,928/743	TN: 37 (30, 45); TE: 46 (38, 53)	24 weeks, 48 weeks	①②
(Torralba et al., 2024)	Retrospective cohort	Spain	2019-2022	PICO1	505 (426/79)	392/113	ALL: 48 (38, 55)	6 months, 12 months	①
(d'Arminio Monforte et al., 2024)	Prospective cohort	Italy	2016–2023	PICO1	2,588 (1,655/933)	2,106/482	TN: 39 (31, 49); TE: 52 (44, 59)	69.8 weeks	②
(Nasreddine et al., 2023)	Prospective cohort	Belgium	2019–2020	PICO1	2,001 (1,593/408)	1,299/702	Median (n): <50 years: 1184; ≥50years: 817	48 weeks	①②
(Mendoza et al., 2023)	Ambispective cohort	Spain	2018–2022	PICO1, 2	131 (119/12)	97/34	TN: 40 (25–47); TE: 49 (40–57)	48 weeks	②
(Kong et al., 2023)	Prospective cohort	China	2022–2023	PICO1	209 (177/32)	195/14	TN: 31.0 (27.5, 36.5); TE: 36.0 (32.0, 43.0)	24 weeks	①②
(Ambrosioni et al., 2022)	Retrospective cohort	Spain	2018–2021	PICO1	1,584 (1,371/213)	1,379/205	ALL: 43 (34, 52)	6 months, 12 months	①
(Yang et al., 2026b)	Retrospective cohort	Asian	2019–2025	PICO2	1,087 (681/406)	1,017/70	DTG/3TC: 40 ± 15; BIC/FTC/TAF: 36 ± 12	48 weeks	①②
(Wu et al., 2026)	Retrospective cohort	China	2020–2023	PICO2	10,971 (7,992/2,979)	9,514/1,457	BIC/FTC/TAF: 36 (29-51); DTG/3TC: 45 (31-61)	12 months	①
(Sun et al., 2026)	Retrospective cohort	China	2020–2023	PICO2	1,445 (901/544)	1,277/168	BIC/FTC/TAF: 36 (30-47); DTG/3TC: 38 (30-53)	12 months	①
(Serrano-Villar et al., 2026)	RCT (open-label)	Spain	NR	PICO2	141 (141/0)	122/19	BIC/FTC/TAF: 45.6 ± 11.2; DTG/3TC: 44.7 ± 11.1	96 weeks	①
(Perez-Valero et al., 2026)	RCT	Spain	NR	PICO2	80 (80/0)	61/19	BIC/FTC/TAF: 48.22 ± 11.6; DTG/3TC: 49.31 ± 9.0	48 weeks	①②
(Martin-Torres et al., 2026)	Retrospective cohort	Spain	2019–2024	PICO2	30 (0/30)	38/2	BIC/FTC/TAF: 28 (24–38); DTG/3TC: 31 (26–34)	48 weeks	①
(Marcelin et al., 2026)	Retrospective cohort	France	2022–2024	PICO2	5,963 (5,963/0)	NR	NR	3 years	①
(Guo et al., 2026)	Retrospective cohort	China	2021–2023	PICO2	194 (0/194)	185/9	BIC/FTC/TAF: 34 (28-40); DTG/3TC: 30 (27-39)	48 weeks	①②
(Ghosn et al., 2026)	RCT	France	NR	PICO2	509 (0/509)	430/79	BIC/FTC/TAF: 33 (18-84); DTG/3TC: 33 (18-73)	48 weeks	①②
(Fan et al., 2026)	Prospective cohort	China	2021–2023	PICO2	212 (0/212)	169/43	BIC/FTC/TAF: 47 ± 14; DTG/3TC: 51 ± 13	48 weeks	①
(Ciccullo et al., 2026)	Retrospective cohort	Italy	2020–2021	PICO2	756 (756/0)	516/240	ALL: 53 (46-59)	48 weeks	②
(Pierone et al., 2025)	Retrospective cohort	US	2020–2022	PICO2	5,839 (5,839/0)	NR	BIC/FTC/TAF: 45 ± 13; DTG/3TC: 46 ± 13	15 months	②
(Liu et al., 2025)	Prospective cohort	China	2021–2022	PICO2	96 (0/96)	96/0	BIC/FTC/TAF: 32 (28-39); DTG/3TC: 32 (29-35)	48 weeks	①
(Lee et al., 2024)	Retrospective cohort	China	2019–2023	PICO2	988 (988/0)	923/65	ALL: 38 (32-46)	48 weeks	①②
(Gan et al., 2024)	Retrospective cohort	China	2020–2022	PICO2	276 (0/276)	205/71	BIC/FTC/TAF: 43 (29-54.5); DTG/3TC: 61 (48-72)	48 weeks	①
(Knobel et al., 2023)	Retrospective cohort	Spain	2019–2022	PICO2	690 (690/0)	602/88	DTG/3TC: 47.7 ± 12.6; BIC/FTC/TAF: 45.1 ± 11.1	48 weeks	①②

TE: treatment-experienced; TN: treatment-naïve; SD: standard deviation; IQR: inter quartile range. Data for age are presented as mean ± SD, median (IQR), or median (range).

①: HIV-RNA <50 copies/mL; ②: Discontinuations.

Among the included studies, Yang 2026, Yokomaku 2025, Kim 2025, Inciarte 2025, Hocqueloux 2025, and Trizzino 2025 are BICSTaR-related cohort studies. Kim 2025 (BICSTaR Korea) overlaps with the Korean subset of Yang 2026 (BICSTaR Asia); therefore, Kim 2025 was not included in the meta-analysis and was presented descriptively only.

### Meta-analysis and descriptive results

#### Virologic suppression rate in TE and TN participants

We first performed a single-group meta-analysis of virologic suppression (HIV-1 RNA <50 copies/mL) for TE and TN populations separately, stratified by follow-up duration (Figure 2). For TE participants, the pooled suppression rate was 95% (95% CI: 91%–98%) at 6 months (1 study), 95% (95% CI: 92%–97%) at 12 months (9 studies, I^2^ = 91%), and 96% (95% CI: 91%–98%) at 24 months (2 studies, I^2^ = 56%). For TN participants, the pooled suppression rate was 84% (95% CI: 67%–95%) at 6 months (1 study), 90% (95% CI: 85%–94%) at 12 months (9 studies, I^2^ = 85%), and 89% (95% CI: 80%–95%) at 24 months (2 studies, I^2^ = 0%). In this analysis, Kim 2025 was not included in the meta-analysis because its data overlap with those of Yang 2026.

**FIGURE 2:**
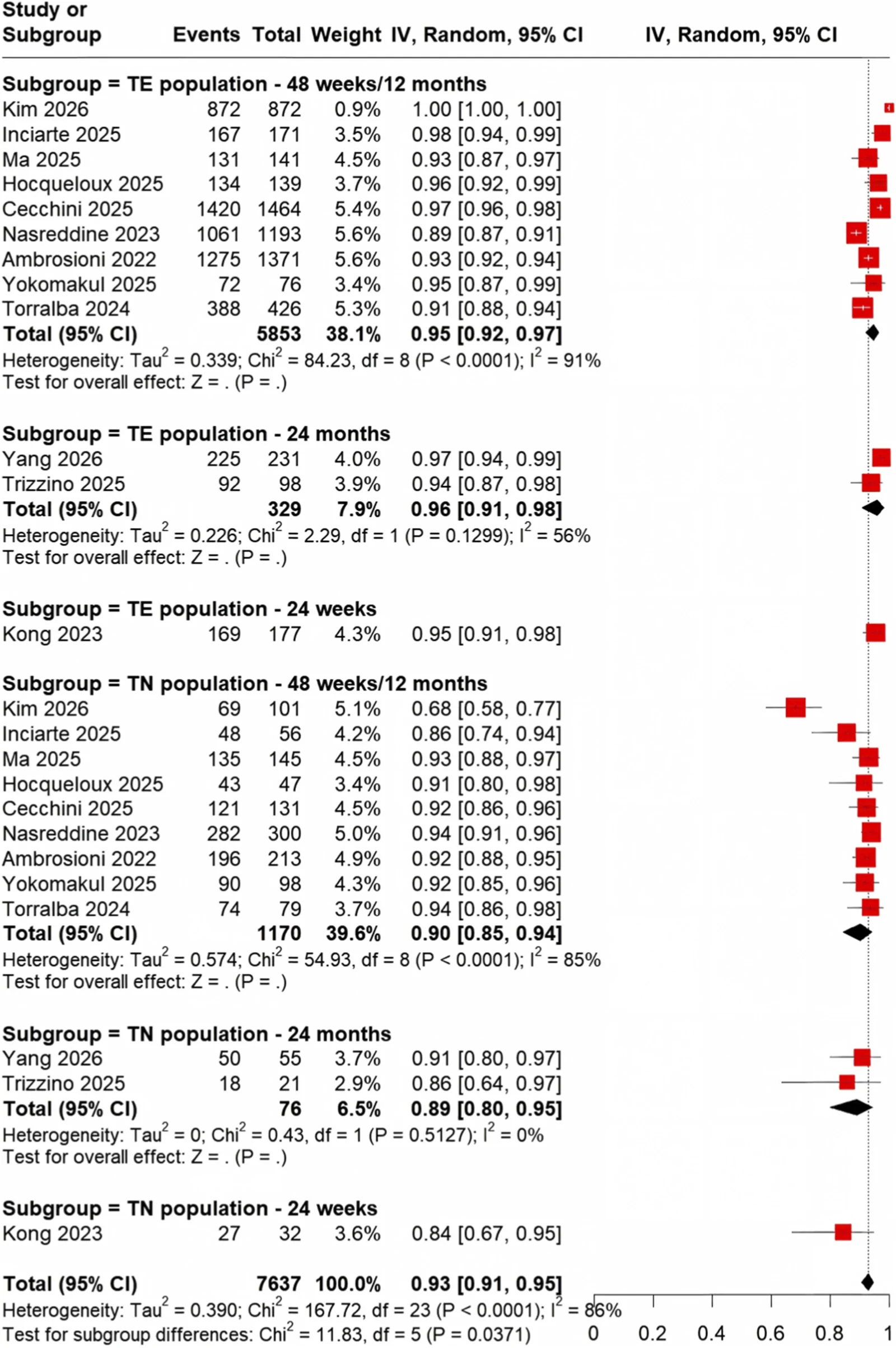
Forest plot of the pooled virologic suppression rate in treatment-experienced and treatment-naïve people.

However, the interpretation of these estimates requires careful consideration of the baseline differences between TE and TN populations. TE participants were predominantly virologically suppressed at the time of switch, whereas TN participants initiated therapy with detectable viremia, representing fundamentally different clinical scenarios. To contextualize these differences and avoid potentially misleading comparative inferences, we summarized the key baseline characteristics of both TE and TN participants across the included studies. As shown in Table 2, TE and TN populations differed substantially across multiple baseline characteristics. Among TE participants, the proportion with baseline HIV-1 RNA < 50 copies/mL ranged from 68.1% to approximately 100% across studies, with most studies reporting rates above 88%. The predominant reason for switching to BIC/FTC/TAF was regimen simplification or optimization (ranging from 32% to 66% across studies), rather than treatment failure or intolerance. In contrast, TN participants universally had detectable viremia at baseline, with median baseline HIV-1 RNA levels ranging from 4.0 to 5.7 copies/mL. Despite these substantial baseline differences, both populations demonstrated high virologic suppression rates following BIC/FTC/TAF treatment. Pooled suppression rates ranged from 91% to 97% for TE participants and from 84% to 91% for TN participants across different follow-up durations. Notably, even in TN participants, the suppression rates at 12 and 24 months exceeded 90% in most studies, indicating that BIC/FTC/TAF is highly effective in achieving viral suppression in the treatment-naïve setting. For TE participants, the consistently high suppression rates (≥93% at all timepoints) further support the regimen’s effectiveness in maintaining virologic control among individuals switching from prior therapies. Taken together, these descriptive estimates indicate that BIC/FTC/TAF achieves favorable virologic outcomes across both populations.

**TABLE 2:** Baseline characteristics and virologic suppression rates of TE and TN participants across included studies.

Study	Sample size for analysis	Baseline VL < 50 (%)	Baseline VL (log10), median (IQR)	Baseline CD4 (cells/μL), median (IQR)	Prior ART duration (years) for TE	Main reason for switch (%) of TE	BIC/FTC/TAF duration	Virologic suppression rate
(Kim et al., 2026)	TE: 872; TN: 101	TE: 100%; TN: NR	TE: 0.00 [0.0, 0.0]; TN: 11.0 [9.8, 12.2]	TE: 645 [470, 880]; TN: 325.0 [186.0, 550.0]	Mean ± SD: 5.7 ± 4.9	New drug availability (98.5%)	48 weeks	TE: 872/872 (100%); TN: 69/101 (68.3%)
(Yang et al., 2026a)	TE: 231; TN: 55	TE: 91.8%; TN: 0%	TE: 1.3 [1.3, 1.3]; TN: 4.5 [4.1, 5.4]	TE: 590 [442, 809]; TN: 325 [190, 476]	3.0 [2.0, 4.0]	Simplification (66%)	24 months	TE: 225/231 (97%); TN: 50/55 (90.9%)
(Inciarte et al., 2025)	TE: 171; TN: 56	TE: 92.8%; TN: 1.6%	TE: 1.48 [1.28, 1.69]; TN: 4.71 [3.70, 5.32]	TE: 773 [597, 955]; TN: 338.0 [147.0, 490.0]	3.0 [2.0, 5.0]	Simplification (43.3%)	12 months	TE: 167/171 (97.7%); TN: 48/56 (85.7%)
(Trizzino et al., 2025)	TE: 98; TN: 21	TE: 89%; TN: 0%	NR	Mean ± SD: TE: 663 ± 389; TN: 176.4 ± 180.9	Mean ± SD: 20.9 ± 9.6	Simplification/optimization (19.8%)	24 months	TE: 92/98 (93.9%); TN: 18/21 (85.7%)
(Ma et al., 2025)	TE: 141; TN: 145	TE: 88.8%; TN: 0%	NR	TE: 333 [242, 499]; TN: 297.0 [120.8, 442.3]	NR	Treatment optimization (34.3%)	48 weeks	TE: 131/141 (92.9%); TN: 135/145 (93.1%)
(Kim et al., 2025)	TE: 37; TN: 29	NR	NR	TE: 572 [427, 714]; TN: 572 [427, 714]	NR	NR	24 months	TE: 37/37 (100%); TN: 29/29 (100%)
(Hocqueloux et al., 2025)	TE: 139; TN: 47	TE: 93%; TN: 0%	TE: 1.3 [1.3, 1.6]; TN: 5.0 [4.1, 5.4]	TE: 659 [419, 928]; TN: 341 [132, 490]	3.0 [2.0, 6.0]	Simplification (54%)	12 months	TE: 134/139 (96.4%); TN: 43/47 (91.5%)
(Cecchini et al., 2025)	TE: 1464; TN: 131	TE: 89%; TN: 1.6%	NR	TE: 621 [436, 843]; TN: 329 [171, 505]	2.0 [1.0, 3.0]	Simplification (54.8%)	48 weeks	TE: 1420/1464 (97.0%); TN: 121/131 (92.4%)
(Kong et al., 2023)	TE: 177; TN: 32	TE: 89.2%; TN: 0%	NR	TE: 430 [291, 572]; TN: 196.5 [46.5, 378.0]	NR	Reduce drug burden (35.6%)	24 weeks	TE: 169/177 (95.5%); TN: 27/32 (84.4%)
(Nasreddine et al., 2023)	TE: 1193; TN: 300	TE: 68.1%; TN: 0%	NR	TE and TN: 567 [367, 793]	TE and TN: 6.0 [0.9, 12.3]	Simplification/pill burden (61.1%)	48 weeks	TE: 1061/1193 (88.9%); TN: 282/300 (94.0%)
(Torralba et al., 2024)	TE: 426; TN: 79	TE: 89%; TN: 2.5%	TE: 1.28 [0, 1.48]; TN: 4.86 [4.0, 5.7]	TE: 670 [466, 891]; TN: 243 [63, 472]	2 [2, 4]	Improvement in tolerance (34.0%)	12 months	TE: 388/426 (91.0%); TN: 74/79 (93.7%)
(Ambrosioni et al., 2026)	TE: 1371; TN: 213	TE: 82%; TN: 0%	TE: <50 (<50–176); TN: 68,500 [19,600, 261,000]	TE: 629 [441, 846]; TN: 357 [194, 496]	2 [1, 4]	Simplification (32%)	12 months	TE: 1,275/1,371 (93.0%); TN: 196/213 (92.0%)
(Yo et al., 2025)	TE: 76; TN: 98	TE: 88%; TN: 0%	TE: 1.28 [1.28, 1.28]; TN: 4.85 [4.46, 5.30]	TE: 572 [427–714]; TN: 297.5 [173.0, 480.0]	2.0 [1.0–3.0]	Simplification (58%)	12 months	TE: 72/76 (94.7%); TN: 90/98 (91.8%)

TE, treatment-experienced; TN, treatment-naïve; VL, viral load; ART, antiretroviral therapy; BIC/FTC/TAF, bictegravir/emtricitabine/tenofovir alafenamide; SD, standard deviation; IQR, interquartile range; NR, not reported.

### Discontinuation rate in TE and TN participants

For discontinuation, we performed exploratory meta-analyses where follow-up durations and reasons for discontinuation were sufficiently homogeneous across studies (Figure 3). For all-cause discontinuation at 48 weeks/12 months, the pooled OR for TE versus TN was 0.81 (95% CI: 0.56–1.17; P = 0.26; I^2^ = 0%), indicating no statistically significant difference between the two groups. At 24 months, the pooled OR was 0.61 (95% CI: 0.29–1.29; P = 0.20; I^2^ = 69%), with no significant difference detected, although the estimate was imprecise due to the limited number of studies. For discontinuation attributed to adverse events at 48 weeks/12 months, the pooled OR was 0.77 (95% CI: 0.43–1.37; P = 0.37; I^2^ = 54%), again showing no significant difference between TE and TN participants. At 24 months, only one study reported adverse event-related discontinuation, with a pooled OR of 1.75 (95% CI: 0.09–34.36; P = 0.71), which should be interpreted with caution due to the very small number of events. In this analysis, Kim 2025 was not included in the meta-analysis because its data overlap with those of Yang 2026; it was therefore presented descriptively only.

**FIGURE 3:**
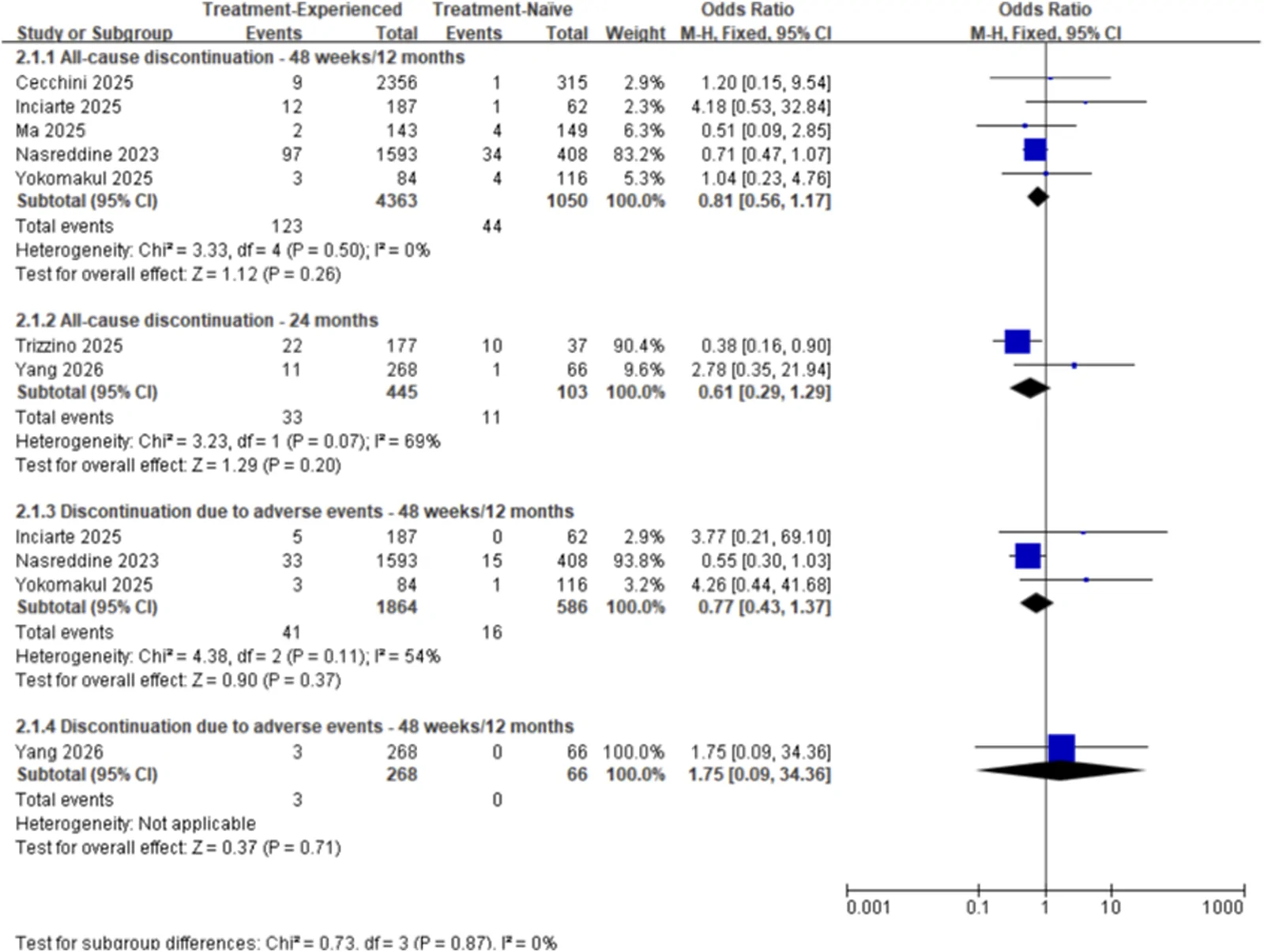
Forest plot of discontinuation comparing treatment-experienced and treatment-naïve individuals.

A detailed breakdown of discontinuation by specific reasons—including patient decision, loss to follow-up, death, virological failure, and others, is provided in Supplementary Table S4. Across all studies and timepoints, discontinuation rates were low in both TE and TN populations, and no consistent pattern of differential risk between the two groups was observed.

#### Virologic suppression rate in BIC/FTC/TAF vs. DTG/3TC

A total of 15 studies reported virologic suppression outcomes for both BIC/FTC/TAF and DTG/3TC regimens (Figure 4). Of these, ten studies were eligible for meta-analysis, comprising three RCTs (two in TE populations and one in TN population) and seven cohort studies (one in TE population and six in TN populations), with a total of 4,851 participants. Because randomized and observational designs differ substantially in confounding structure, treatment allocation, and evidentiary strength, we prioritized design-specific and population-specific estimates rather than the mixed pooled estimate. Among TE populations, the pooled OR was 1.04 (95% CI: 0.34–3.16; I^2^ = 0%) for RCTs and 0.92 (95% CI: 0.42–2.02) for the single cohort study (Knobel et al., 2023); among TN populations, the pooled OR was 1.46 (95% CI: 0.80–2.66) for the single RCT (Ghosn et al., 2026) and 0.98 (95% CI: 0.70–1.39; I^2^ = 0%) across six cohort studies. No statistically significant difference was detected in any of these stratified analyses. Test for subgroup differences was not significant (P = 0.71, I^2^ = 0%), indicating that the comparative effect did not vary by study design or treatment history. When all ten studies were combined regardless of design, the exploratory mixed pooled OR was 1.06 (95% CI: 0.81–1.39; P = 0.66; I^2^ = 0%).

**FIGURE 4:**
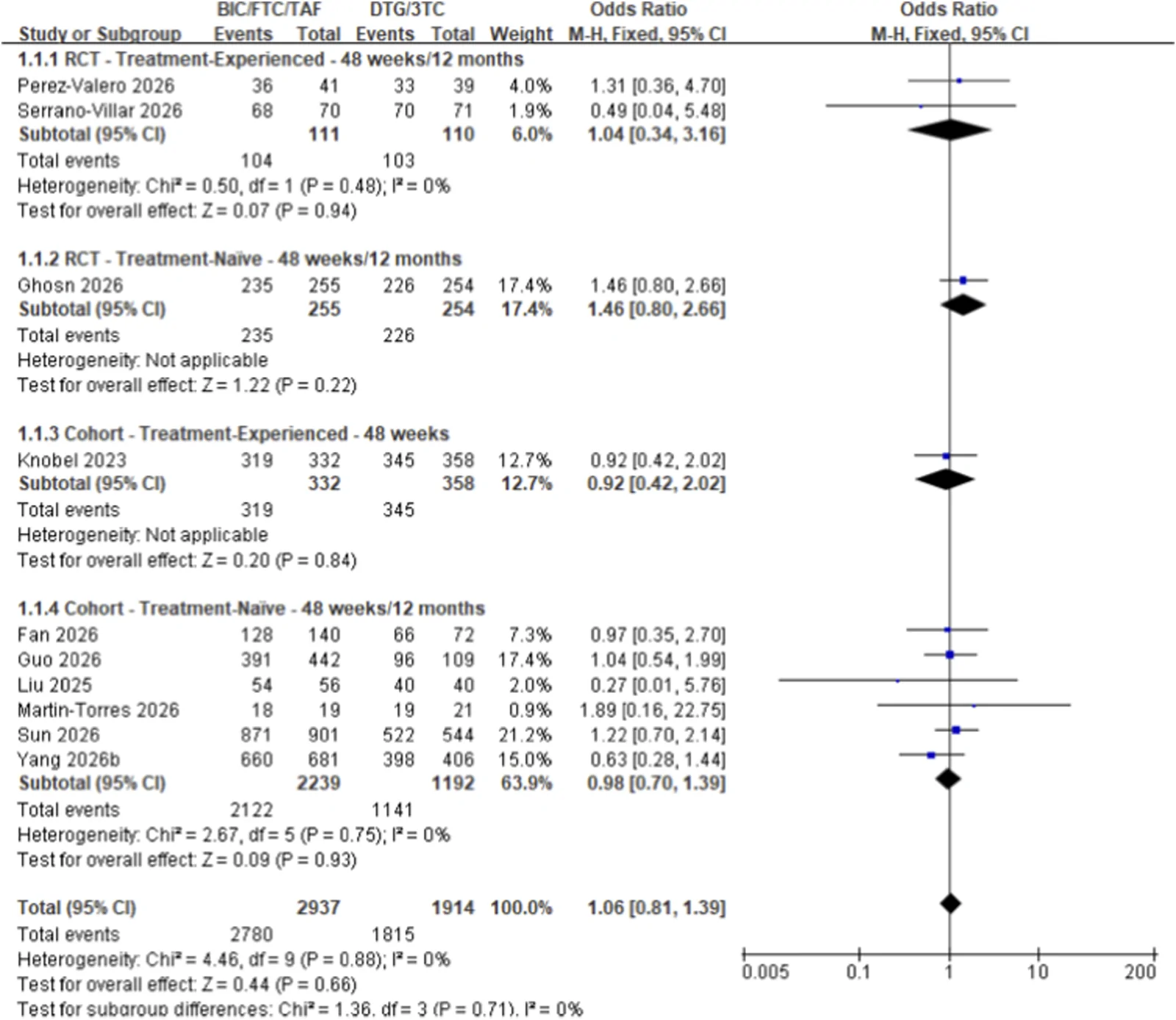
Forest plot of virologic suppression in BIC/FTC/TAF versus DTG/3TC.

The remaining five studies could not be pooled in the meta-analysis due to substantial heterogeneity in study populations, outcome definitions, or severe channeling bias; these studies are summarized descriptively in Table 3. Wu 2026 was excluded because it reported adjusted incidence rate ratios (aIRRs) rather than odds ratios, and the original study did not report extractable 2 × 2 event counts for the 12-month virologic suppression outcome. Marcelin 2026 reported virologic failure rates rather than conventional suppression rates, rendering the indirectly derived effect estimate non-commensurate with the primary outcome. Gan 2024, Lee 2024, and Mendoza 2023 were excluded due to severe baseline imbalances between treatment groups, unavailable group-specific outcome data, or heterogeneous populations with imbalanced composition. Notably, the individual effect estimates from these five studies ranged from 0.41 to 1.47, four of the five studies had confidence intervals crossing the null, while one (Lee 2024) did not.

**TABLE 3:** Summary of studies excluded from the pooled analysis of virologic suppression (BIC/FTC/TAF vs. DTG/3TC).

Study	Reason for not including in the meta-analysis	Outcome	Follow-up	BIC/FTC/TAF (r/n, %)	DTG/3TC (r/n, %)	Estimate, 95% CI
(Wu et al., 2026)	Reported adjusted incidence rate ratio (aIRR) rather than odds ratio (OR); original data presented as cumulative incidence rather than extractable 2 × 2 event counts	Virologic suppression–TN population	12 months	84.2%	87.6%	aIRR = 1.00 (0.95–1.05)
(Marcelin et al., 2026)	The outcome measure was virologic failure rate. The indirectly derived suppression rate was not commensurate with the definitions or statistical approaches used in the other included studies	Virologic failure rate–TE population	NR	203/3383 (6.0%)	129/2580 (5.0%)	OR = 1.21 (0.97–1.52)
(Gan et al., 2024)	Severe channeling bias: BIC/FTC/TAF group significantly younger (43 vs. 61 years), higher proportion with VL > 500K (22.9% vs. 9.7%), and lower prevalence of hypertension and diabetes; groups not comparable	Virologic suppression–TN population	48 weeks	108/131 (82.4%)	129/145 (89.0%)	OR = 0.58 (0.29–1.15)
(Lee et al., 2024)	Baseline characteristics not stratified by treatment group; unable to assess comparability; no separate outcome data by regimen for key endpoints	Virologic suppression–TE population	48 weeks	456/480 (95.0%)	497/508 (97.8%)	OR = 0.41 (0.21–0.79)
(Mendoza et al., 2023)	Heterogeneous population (TN and TE pooled); imbalanced TN/TE proportions between groups; HBV coinfection and prior regimen count differed significantly; follow-up duration differed between groups	Virologic suppression–TE population	48 weeks	114/119 (95.8%)	126/134 (94.0%)	OR = 1.47 (0.51–4.22)

BIC/FTC/TAF, bictegravir/emtricitabine/tenofovir alafenamide; DTG/3TC, dolutegravir/lamivudine; OR, odds ratio; CI, confidence interval; TE, treatment-experienced; TN, treatment-naïve; VL, viral load; HBV, hepatitis B virus.

#### Discontinuation rate in BIC/FTC/TAF vs. DTG/3TC

A total of eight studies reported discontinuation outcomes comparing BIC/FTC/TAF and DTG/3TC (Figure 5). Given the substantial heterogeneity in study populations, treatment indications, and follow-up durations, only two meta-analyses could be performed. In the treatment-naïve subgroup at 48 weeks, a meta-analysis of two cohort studies (Yang et al., 2026b; Guo et al., 2026) reported all-cause discontinuation rates, showing no significant difference between BIC/FTC/TAF and DTG/3TC (pooled OR = 1.02, 95% CI: 0.70–1.49; P = 0.93; I^2^ = 0%). For discontinuation due to adverse events in the same population, a meta-analysis of two studies (Yang et al., 2026b; Ghosn et al., 2026) similarly showed no significant difference between regimens (pooled OR = 1.11, 95% CI: 0.55–2.22; P = 0.78; I^2^ = 0%). In contrast, among treatment-experienced populations, a single RCT (Perez-Valero et al., 2026) at 48 weeks (OR = 0.76, 95% CI: 0.21–2.74; P = 0.68) and the large OPERA cohort study (Pierone et al., 2025) at a median follow-up of 15–16 months (OR = 0.87, 95% CI: 0.76–1.00; P = 0.04) provided data for this subgroup. For discontinuation due to adverse events, Perez-Valero 2026 (OR = 1.30, 95% CI: 0.27–6.21; P = 0.74) and Pierone 2025 (OR = 0.59, 95% CI: 0.30–1.15; P = 0.12) both showed no significant difference in treatment-related or adverse event-related discontinuations among treatment-experienced populations.

**FIGURE 5:**
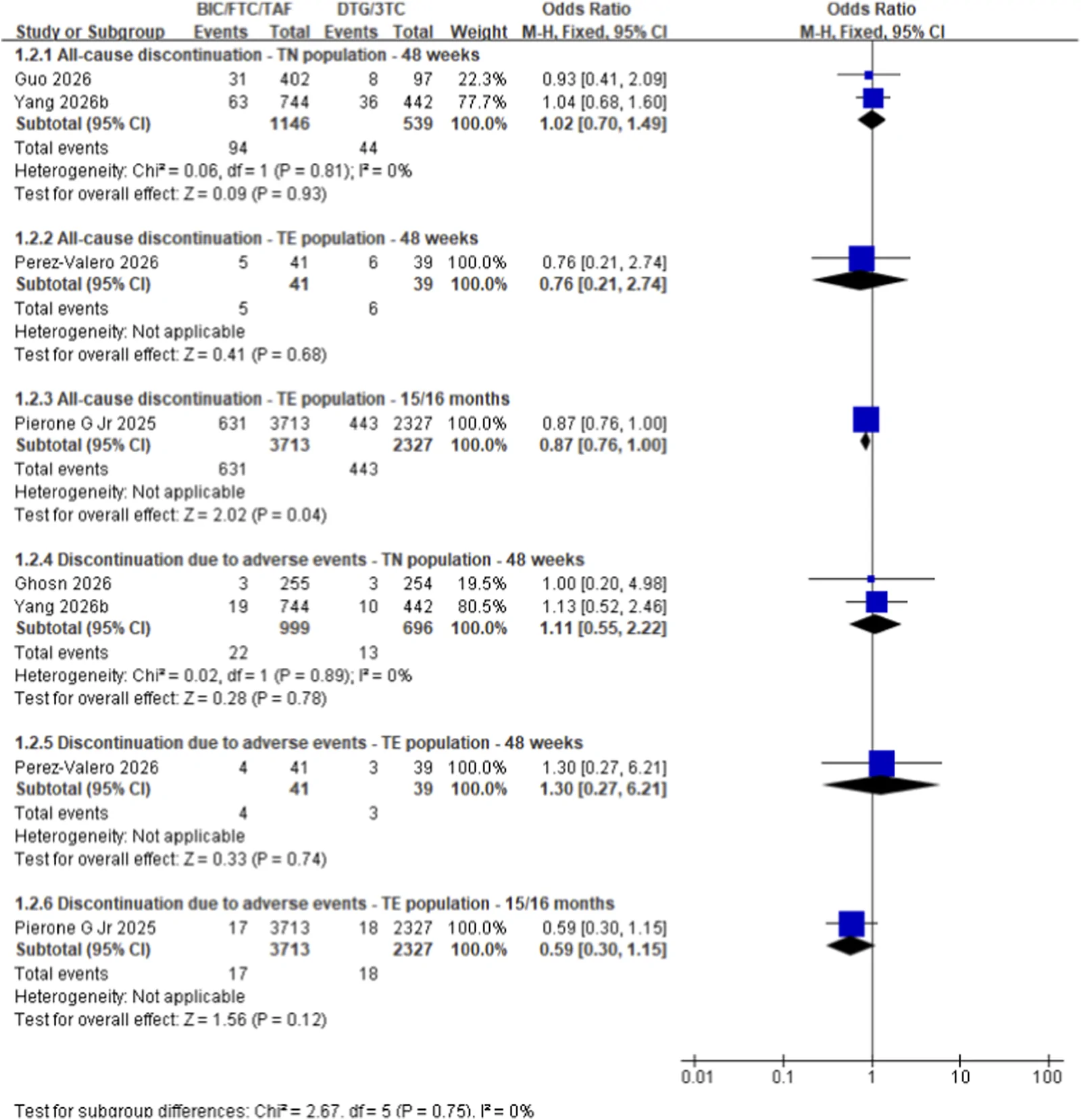
Forest plot of discontinuation in BIC/FTC/TAF versus DTG/3TC.

Detailed breakdowns of discontinuation by specific reasons are presented in Supplementary Table S5. Across all studies and populations, the most commonly reported reasons for discontinuation included adverse events (particularly weight gain, gastrointestinal symptoms, and neuropsychiatric effects), loss to follow-up, patient or provider preference, and regimen simplification or switching. Notably, weight gain was more frequently cited as a reason for discontinuation in the BIC/FTC/TAF group (1.5% in Lee 2024; 0.7% in Ciccullo 2026), whereas neuropsychiatric adverse events were reported in both groups at low frequencies. The OPERA cohort (Pierone et al., 2025) provided the largest dataset, showing that non-treatment-related discontinuations (e.g., loss to follow-up, treatment gaps, provider choice) accounted for the majority of all-cause discontinuations in both groups (14.1% for BIC/FTC/TAF vs. 15.4% for DTG/3TC), whereas treatment-related discontinuations were rare (1.0% vs. 1.7%). Overall, the stratified meta-analyses and descriptive summaries indicate that discontinuation rates are low in both groups across different populations, with no statistically significant difference detected in most comparisons, with the possible exception of a modest all-cause discontinuation advantage for BIC/FTC/TAF observed in the OPERA cohort.

### Sensitivity analysis

To assess the robustness of the pooled results, we performed leave-one-out sensitivity analyses by sequentially omitting each individual study from each meta-analysis. As shown in Supplementary Table S6, For the TE versus TN comparisons within BIC/FTC/TAF, the leave-one-out analyses for all-cause discontinuation at 48 weeks/12 months yielded ORs ranging from 0.73 to 1.30 (overall OR = 0.81, 95% CI: 0.56–1.17); at 24 months, ORs ranged from 0.38 to 2.78 (overall OR = 0.61, 95% CI: 0.29–1.29); and for discontinuation due to adverse events at 48 weeks/12 months, ORs ranged from 0.65 to 4.03 (overall OR = 0.77, 95% CI: 0.43–1.37). In all cases, the 95% confidence intervals crossed the null value of 1.0, confirming that no individual study disproportionately influenced the pooled estimates.

For the BIC/FTC/TAF versus DTG/3TC comparisons, the leave-one-out analyses for virologic suppression in the treatment-naïve cohort subgroup (six studies) yielded ORs ranging from 0.87 to 1.09 (overall OR = 0.98, 95% CI: 0.70–1.39); for all-cause discontinuation in the treatment-naïve population at 48 weeks (two studies), ORs ranged from 0.93 to 1.04 (overall OR = 1.02, 95% CI: 0.70–1.49); and for discontinuation due to adverse events in the same population (two studies), ORs ranged from 1.00 to 1.13 (overall OR = 1.11, 95% CI: 0.55–2.22). For the RCT subgroup in treatment-experienced populations, excluding either study yielded ORs of 0.49 or 1.31 (overall OR = 1.04, 95% CI: 0.34–3.16). All confidence intervals crossed the null, indicating that the null findings were not driven by any single study.

### Certainty of evidence

We assessed the certainty of evidence for key comparative outcomes using the GRADE framework (Table 4), which is organized according to the two research questions of this meta-analysis. For the comparison between TE and TN participants receiving BIC/FTC/TAF, three outcomes were evaluated: all-cause discontinuation at 48 weeks/12 months (five observational studies), all-cause discontinuation at 24 months (three observational studies), and discontinuation due to adverse events at 48 weeks/12 months (three observational studies). The certainty of evidence was rated as low for all three outcomes, with downgrading primarily attributable to the observational study designs and imprecision of the effect estimates.

**TABLE 4:** Summary of findings and GRADE evidence quality for key comparative outcomes.

Outcome and follow-up	Patients (studies), N	Relative effect (95% CI)	Absolute effects (95% CI)			Certainty
			Control group	Observation group	Difference	
			TN population	TE population	​	
All-cause discontinuation - 48 weeks/12 months	5413 (5 non-randomised studies)	OR = 0.81 (0.56–1.17)	42 per 1,000	34 per 1,000 (24–49)	8 fewer per 1,000 (from 18 fewer to 7 more)	⊕○○ Low
All-cause discontinuation - 24 months	548 (2 non-randomised studies)	OR = 0.61 (0.29–1.29)	107 per 1,000	68 per 1,000 (34–134)	39 fewer per 1,000 (from 73 fewer to 27 more)	⊕⊕○○ Low
Discontinuation due to adverse events - 48 weeks/12 months	2450 (3 non-randomised studies)	OR = 0.77 (0.43–1.37)	27 per 1,000	21 per 1,000 (12–37)	6 fewer per 1,000 (from 15 fewer to 10 more)	⊕⊕○○ Low
​	​	​	DTG/3TC	BIC/FTC/TAF	​	​
Virologic suppression-overall	4851 (10 mixed studies)	OR = 1.06 (0.81–1.39)	948 per 1,000	951 per 1,000 (937–962)	3 more per 1,000 (from 11 fewer to 14 more)	⊕○○○ Very low^a^
Virologic suppression- TE population - 48 weeks/12 months	221 (2 RCTs)	OR = 1.04 (0.34–3.16)	936 per 1,000	939 per 1,000 (833–979)	2 more per 1,000 (from 103 fewer to 43 more)	⊕⊕⊕○ Moderate^b^
Virologic suppression - TN population - 48 weeks/12 months	3431 (6 non-randomised studies)	OR = 0.98 (0.70–1.39)	957 per 1,000	956 per 1,000 (940–969)	1 fewer per 1,000 (from 17 fewer to 12 more)	⊕⊕○○ Low
All-cause discontinuation - TN population - 48 weeks	1685 (2 non-randomised studies)	OR = 1.02 (0.70–1.49)	82 per 1,000	83 per 1,000 (59–117)	1 more per 1,000 (from 23 fewer to 35 more)	⊕⊕○○ Low
Discontinuation due to adverse events -TN population - 48 weeks	1695 (2 non-randomised studies)	OR = 1.11 (0.55–2.22)	19 per 1,000	21 per 1,000 (10–42)	2 more per 1,000 (from 8 fewer to 22 more)	⊕⊕○○ Low

CI, confidence interval; OR, odds ratio; TE, treatment-experienced; TN, treatment-naïve; RCT, randomized controlled trial.

a Mixed research design.

b An overly wide confidence interval.

For the comparison between BIC/FTC/TAF and DTG/3TC, five outcomes were assessed: overall virologic suppression (ten mixed studies, including three RCTs and seven cohort studies), virologic suppression in the treatment-experienced RCT subgroup (two RCTs), virologic suppression in the treatment-naïve cohort subgroup (six observational studies), all-cause discontinuation in the TN population at 48 weeks (two cohort studies), and discontinuation due to adverse events in the TN population at 48 weeks (two studies, including one RCT and one cohort study). The certainty of evidence varied across these outcomes: the overall estimate was rated as very low due to the mixed study designs and imprecision; the treatment-experienced RCT subgroup achieved moderate certainty, downgraded solely for imprecision; and the treatment-naïve cohort subgroup for virologic suppression, all-cause discontinuation, and adverse event-related discontinuation were all rated as low certainty, downgraded for observational designs and imprecision. Overall, the GRADE assessment indicates that the certainty of evidence is limited primarily by the predominance of observational studies and imprecise effect estimates, underscoring the need for additional well-powered randomized controlled trials to strengthen the evidence base for these comparative effectiveness findings.

## Discussion

This study systematically compared the efficacy of BIC/FTC/TAF between TE and TN people with HIV-1. The key findings showed that BIC/FTC/TAF achieved high virologic suppression rates in both TE and TN populations, with low discontinuation rates observed in both groups. Treatment discontinuation rates were low in both groups, with pooled estimates of approximately 3% across time points. These results indicate that BIC/FTC/TAF is highly effective in both TE and TN populations, with low discontinuation rates observed across time points, supporting its use across different treatment settings.

The high virologic suppression rates observed in both TE and TN populations are consistent with the known efficacy of BIC/FTC/TAF. Because TE and TN populations differ fundamentally in baseline virologic status and treatment goals, their suppression rates should be interpreted descriptively and not compared directly. First, TE individuals have already integrated long-term ART into their daily routines and may demonstrate more consistent medication-taking habits, whereas TN individuals require a period of adaptation to lifelong treatment (Li et al., 2026). Second, TE participants were generally older than TN participants across most included studies; older age has been associated with better adherence in some HIV cohorts, possibly due to more stable lifestyles and established healthcare relationships (Ettenhofer et al., 2009). Third, a ‘healthy survivor’ effect may also play a role, as TE individuals represent a population that has remained engaged in care, potentially reflecting a better prognosis independent of the specific regimen (Hodgkinson et al., 2020). Importantly, the absolute suppression rates were excellent in both groups, which supports current guideline recommendations for BIC/FTC/TAF as a first-line option (Mezzogori et al., 2025; Al-Hayani et al., 2026).

The low treatment discontinuation rates observed in both TE and TN populations (approximately 2%–3% across time points) highlight the favorable acceptability and persistence of BIC/FTC/TAF in clinical practice. Unlike older regimens associated with renal toxicity, bone mineral density loss, or neuropsychiatric adverse events, BIC/FTC/TAF has consistently demonstrated a high barrier to discontinuation across diverse populations (Chivite et al., 2024; Passerotto et al., 2024). The lack of difference between groups is clinically reassuring for several reasons. First, it suggests that TN patients do not experience more frequent or more severe adverse events during therapy initiation compared to experienced patients, who may have previously tolerated other regimens (Kong et al., 2023). Second, the low discontinuation rate in both groups is substantially lower than those reported for some other integrase strand transfer inhibitor-based regimens, such as elvitegravir/cobicistat-based combinations, in real-world settings (De Castro et al., 2023). Third, the absence of heterogeneity (I^2^ = 0%) in this comparison strengthens the robustness of this finding. Taken together, these results indicate that discontinuation is unlikely to be a major barrier to BIC/FTC/TAF use in either TN or TE individuals, supporting its role as a versatile option across the HIV treatment cascade.

In design-stratified analyses, no statistically significant difference in virologic suppression was detected between BIC/FTC/TAF and DTG/3TC in either RCTs or cohort studies. The exploratory mixed pooled estimate (OR = 1.06, 95% CI: 0.81–1.39) was rated as very low certainty because it combines randomized and observational evidence, and should therefore be interpreted with caution. No statistical differences were also observed in the treatment discontinuation (all-cause discontinuation and the discontinuation due to adverse events). These findings suggest that no large difference was detected between the two regimens in our analysis. However, the absence of a statistically significant difference should not be interpreted as proof of equivalence, as our meta-analysis was conducted within a superiority framework without a predefined non-inferiority margin. These findings are consistent with recent large-scale real-world evidence. In a multicenter retrospective cohort study of 13,895 treatment-naïve individuals across 19 centers in China, Wu et al. reported that DTG/3TC was associated with a similar incidence of virologic suppression compared to BIC/FTC/TAF (adjusted Incidence Rate Ratio = 1.00, 95% CI: 0.95–1.05), although they observed smaller CD4 count and CD4/CD8 ratio increases and a 37% higher all-cause mortality rate with DTG/3TC (Wu et al., 2026). Our findings on viral suppression align with this observation, though our analysis was not powered to assess mortality differences. Additionally, a large French observational study by Marcelin et al. involving 6,523 individuals with at least one prior regimen found that the prevalence of virologic failure was 6% for BIC/FTC/TAF and 5% for DTG/3TC, while treatment-emergent resistance-associated mutations at virologic failure were more common with DTG/3TC (15%) than with BIC/FTC/TAF (3%) (Marcelin et al., 2026). This resistance disparity, though not directly assessed in our study, may have implications for long-term durability, particularly in individuals with prior treatment experience or suboptimal adherence, and warrants further investigation.

We observed low significant heterogeneity in the TE vs. TN comparison for all-cause discontinuation (I^2^ = 0%) and minimal heterogeneity in the BIC/FTC/TAF vs. DTG/3TC comparison for virologic suppression and discontinuation (I^2^ = 0%), supporting the robustness of these findings. In contrast, substantial statistical heterogeneity was observed in the pooled virologic suppression analyses for both TE (I^2^ = 91%) and TN (I^2^ = 85%) populations, which limits the precision of pooled estimates. These high I^2^ values likely reflect the considerable clinical and methodological variability across studies. Several factors likely contributed to this heterogeneity. First, study-level variability was considerable, including differences in geographic regions, sample sizes, and treatment durations. Second, the definition of “treatment-experienced” may have varied across studies, including differences in prior regimen types, duration of prior suppression, and reasons for switching. Third, outcome measurement timing differed, and although subgroup analyses were performed by duration, residual heterogeneity may remain due to inconsistent reporting windows. In the descriptive analyses by follow-up duration, both TE and TN participants showed high pooled suppression rates across all time points (TE: 93%–97%; TN: 84%–91%). For treatment discontinuation, minimal heterogeneity was observed across all time points, further supporting the robustness of this finding. It is noteworthy that two large observational studies, Wu et al. (total cohort n = 13,895; analytic sample for virologic suppression n = 10,971) and Marcelin et al. (overall cohort n = 6,523; BIC/FTC/TAF n = 3,383 and DTG/3TC n = 2,580), contributed substantially to the comparative analysis between BIC/FTC/TAF and DTG/3TC. These two large observational studies were not included in the primary pooled analysis for the methodological reasons described above, but their findings were broadly consistent with our conclusion of no statistically significant difference in virologic suppression between BIC/FTC/TAF and DTG/3TC. Leave-one-out sensitivity analyses based on the primary analysis dataset showed that the pooled estimates remained stable.

Our findings align with and extend previous meta-analyses on BIC/FTC/TAF. Chivite et al. conducted a systematic review and meta-analysis of real-world data, reporting high effectiveness and low discontinuation rates in both TN and TE populations, but did not perform a direct statistical comparison between the two groups (Chivite et al., 2024). Similarly, Chen et al. demonstrated favorable efficacy and safety of BIC/FTC/TAF among people living with HIV, primarily based on individual arm-level data from clinical trials (Chen et al., 2021). Furthermore, the low discontinuation rates observed in our study are consistent with the findings from previous phase 3 trials and real-world cohorts (Kityo et al., 2025; Trottier et al., 2025), further supporting the acceptability of BIC/FTC/TAF in clinical practice. More recently, Curteis et al. conducted a Bayesian network meta-analysis (Curteis et al., 2025) comparing the safety of BIC/FTC/TAF with other ART regimens in TN people with HIV. Across 19 RCTs, BIC/FTC/TAF showed a favourable safety profile, particularly for discontinuation due to adverse events, treatment-related adverse events, and nausea, and was non-inferior to all comparators for grade 3/4 treatment-emergent adverse events, diarrhoea, and all-cause discontinuation. However, that analysis was restricted to RCTs and TN individuals, focused on safety rather than virologic suppression, and did not evaluate DTG/3TC as a dedicated comparator. Our study therefore complements these findings by comparing BIC/FTC/TAF with DTG/3TC for virologic suppression and discontinuation in both TN and TE populations, including real-world cohort evidence.

The findings of this meta-analysis have several practical implications. First, clinicians can confidently prescribe BIC/FTC/TAF to both TN and TE individuals, with the expectation of high virologic suppression rates and low discontinuation rates. Second, the high suppression rates observed in TN individuals support the use of BIC/FTC/TAF as a first-line option. In TE individuals, the high suppression rates observed after switching are consistent with the regimen’s effectiveness in maintaining virologic control. These findings support the use of BIC/FTC/TAF across both treatment settings. Last, it is also important to note that all included studies were conducted in Asia, Europe, and the Americas, with no data from Africa, particularly Sub-Saharan Africa. This geographic gap is noteworthy because Sub-Saharan Africa carries the highest global burden of HIV, where non-B HIV-1 subtypes (e.g., subtype C) predominate and may influence virologic responses to integrase strand transfer inhibitors. Moreover, region-specific factors such as healthcare infrastructure, antiretroviral drug supply chain stability, and patient retention in care could substantially affect real-world effectiveness and discontinuation rates. While our analysis included large Chinese cohorts that provide important evidence for Asian populations, these findings may not be directly generalizable to African settings. Additionally, the vast majority of participants across the included studies were male, reflecting the demographic characteristics of many HIV cohorts in the regions studied. This marked underrepresentation of women limits the generalizability of our findings to female populations and to pregnant or breastfeeding individuals, in whom pharmacokinetic and safety profiles may differ. Future studies should actively enroll more women and ensure adequate representation of diverse populations to improve the generalizability of evidence across sexes and geographic regions.

Several limitations warrant consideration. First, although we included both observational cohort studies and RCTs in our meta-analysis, the majority of the included studies were observational in design. This predominance of observational evidence may introduce selection bias and confounding by indication, and the relatively small number of RCTs limits the strength of our comparative conclusions. Second, substantial heterogeneity in the virologic suppression analysis limits the precision of pooled estimates, and our subgroup analyses could only partially explain this variability. Third, we did not assess other important outcomes, such as resistance-associated mutations, specific adverse events (e.g., renal, bone, neuropsychiatric), or quality of life measures. Moreover, adherence, prior treatment duration and reasons for switching are key patient-level determinants of virologic response, but these variables were not uniformly reported across the included studies and therefore could not be incorporated into our analysis, which further limits the interpretability of our findings. Meanwhile, for the observational comparisons, adjusted effect estimates were not reported for the virologic suppression or discontinuation outcomes in the included cohort studies; therefore, unadjusted event counts were used. This may have introduced residual confounding, and the resulting estimates should be interpreted with caution. This lack of adjustment for important patient-level confounders also contributed to the downgrading of evidence certainty in our GRADE assessment, particularly for observational study estimates, as unmeasured confounding may have influenced the observed effect sizes. Fourth, most studies had follow-up durations of 12 months or less, with limited long-term (≥24 months) data. Fifth, the definition of “treatment-experienced” varied across studies, and we could not perform subgroup analyses based on prior regimen type or virologic suppression status at switch. Future directions. Future research should focus on the large, prospective cohort studies with standardized definitions of treatment experience and longer follow-up periods (≥48 months), as well as investigations of patient-level factors (e.g., adherence measured by drug levels or electronic monitors) that mediate the relationship between treatment history and virologic outcomes. Sixth, all included studies originated from Asia, Europe, and the Americas; the absence of data from Africa, particularly Sub-Saharan Africa, limits the generalizability of our findings to settings with the highest HIV burden and distinct epidemiological and socioeconomic contexts.

## Conclusion

This meta-analysis demonstrates that BIC/FTC/TAF achieves high virologic suppression rates and low treatment discontinuation rates in both TE and TN people with HIV-1, supporting its continued use as a preferred option across different treatment settings. No significant difference was observed in treatment discontinuation between TE and TN groups, suggesting that treatment discontinuation is similarly low across diverse patient populations. Furthermore, in design-stratified analyses, BIC/FTC/TAF and DTG/3TC demonstrated no statistically significant difference in virologic suppression or treatment discontinuation rates in our analysis, suggesting that both regimens are viable guideline-preferred options. The choice between them should be individualized based on patient characteristics, including prior treatment history, adherence potential, comorbidity profile, and metabolic risk considerations.

## Data Availability

The original contributions presented in the study are included in the article/Supplementary Material, further inquiries can be directed to the corresponding authors.
