# Supplementary material for: Bictegravir/emtricitabine/tenofovir alafenamide for HIV: a meta-analysis of efficacy across treatment-naïve and experienced populations and comparative effectiveness versus dolutegravir/lamivudine
Source: Front Pharmacol. 2026 Oct 30;17:1935865. doi: 10.3389/fphar.2026.1935865 (PMC13759725; doi:10.3389/fphar.2026.1935865)
Supplement: Supplementary file 1 [file Table1.docx]

**Table S1. The search strategies and results of the main database**

| PubMed | |
| --- | --- |
| #1 | ("bictegravir emtricitabine tenofovir alafenamide drug combination"[Supplementary Concept] OR "bictegravir emtricitabine tenofovir alafenamide drug combination"[All Fields] OR "bictegravir emtricitabine tenofovir alafenamide"[All Fields] OR "b f taf"[All Fields] OR "bic ftc taf"[All Fields]) |
| #2 | ("hiv"[MeSH Terms] OR "hiv"[All Fields] OR ("human"[All Fields] AND "immunodeficiency"[All Fields] AND "virus"[All Fields]) OR "human immunodeficiency virus"[All Fields] OR ("hiv"[MeSH Terms] OR "hiv"[All Fields]) OR (("people s"[All Fields] OR "peopled"[All Fields] OR "peopling"[All Fields] OR "persons"[MeSH Terms] OR "persons"[All Fields] OR "people"[All Fields] OR "peoples"[All Fields]) AND ("lived"[All Fields] OR "lives"[All Fields] OR "living"[All Fields] OR "livings"[All Fields]) AND ("hiv"[MeSH Terms] OR "hiv"[All Fields])) OR "PLWH"[All Fields]) |
| #3 | #1 AND #2 421 |
| Web of Science | |
| #1 | (((TS=(bictegravir/emtricitabine/tenofovir alafenamide)) OR TS=("B/F/TAF") OR TS=("BIC/FTC/TAF") OR TS=(bictegravir) OR TS=("BIC") AND TS=(emtricitabine) AND TS=(tenofovir alafenamide)) |
| #2 | ((TS=(human immunodeficiency virus)) OR TS=(HIV) OR TS=("People living with HIV") OR TS=(PLWH))) |
| #3 | #1 AND #2 633 |
| Embase | |
| #1 | ('human immunodeficiency virus':ti,ab OR 'hiv':ti,ab OR 'plwh':ti,ab) |
| #2 | ('b/f/taf':ti,ab OR 'bictegravir/emtricitabine/tenofovir alafenamide':ti,ab OR 'bic/ftc/taf':ti,ab) |
| #3 | #1 AND #2 1068 |
| Supplementary search | |
| 1 | ClinicalTrials.gov (<https://clinicaltrials.gov/>) 123 |
| 2 | WHO ICTRP (<https://trialsearch.who.int/>) 167 |

**Table S2. Risk of bias assessment for randomized controlled trials using the RoB 2.0 tool**

| **Domain** | **Perez-Valero 2026 (MIND)** | **Serrano-Villar 2026 (INSTINCT)** | **Ghosn 2026 (VOGUE)** |
| --- | --- | --- | --- |
| Randomization process | Low | Low | Low |
| Deviations from intended interventions | Low | Some concerns | Some concerns |
| Missing outcome data | Low | Low | Low |
| Measurement of the outcome | Low | Some concerns | Some concerns |
| Selection of the reported result | Low | Low | Low |
| Overall | Low | Some concerns | Some concerns |

**Table S3. The quality evaluation results of cohort studies**

| **Study** | **①**  **Representativeness of the exposed cohort** | **②**  **Selection of the non-exposed cohort** | **③**  **Ascertainment of exposure** | **④**  **Demonstration that outcome of interest was not present at start of study** | **⑤**  **Comparability of cohorts on the basis of the design or analysis** | **⑥**  **Assessment of outcome** | **⑦**  **Was follow-up long enough for outcomes to occur** | **⑧**  **Adequacy of follow up of cohorts** | **Total score** |
| --- | --- | --- | --- | --- | --- | --- | --- | --- | --- |
| Yang 2026 | ★ | ★ | ★ | ★ | ★★ | ★ | ★ | ★ | 9 |
| Kim 2026 | ★ | ★ | ★ | ★ | ★★ | ★ | ★ | ★ | 9 |
| YokomakuI 2025 | ★ | ★ | ★ | ★ | ★★ | ★ | ★ | ★ | 9 |
| Trizzino 2025 | ★ | ★ | — | ★ | ★ | — | ★ | ★ | 7 |
| Ma 2025 | ★ | ★ | — | ★ | ★★ | ★ | ★ | ★ | 8 |
| Kim 2025 | ★ | ★ | ★ | ★ | ★ | ★ | ★ | ★ | 9 |
| Inciarte 2025 | ★ | ★ | — | ★ | ★★ | ★ | ★ | ★ | 8 |
| Hocqueloux 2025 | ★ | ★ | ★ | ★ | ★★ | ★ | ★ | ★ | 9 |
| Cecchini 2025 | ★ | ★ | — | ★ | ★★ | ★ | ★ | ★ | 8 |
| Torralba 2024 | ★ | ★ | ★ | ★ | ★★ | ★ | ★ | ★ | 9 |
| Monforte 2024 | ★ | ★ | ★ | ★ | ★★ | ★ | ★ | ★ | 9 |
| Nasreddine 2023 | ★ | ★ | ★ | ★ | ★★ | ★ | ★ | — | 8 |
| Mendoza 2023 | ★ | ★ | ★ | ★ | ★★ | ★ | ★ | ★ | 9 |
| Kong 2023 | ★ | ★ | ★ | ★ | ★★ | ★ | ★ | — | 8 |
| Ambrosioni 2022 | ★ | ★ | ★ | ★ | ★★ | ★ | ★ | — | 8 |
| Yang 2026b | ★ | ★ | ★ | ★ | ★★ | ★ | ★ | ★ | 9 |
| Wu 2026 | ★ | ★ | ★ | ★ | ★★ | ★ | ★ | ★ | 9 |
| Sun 2026 | ★ | ★ | ★ | ★ | ★★ | ★ | ★ | ★ | 9 |
| Marcelin 2026 | ★ | ★ | ★ | ★ | ★★ | ★ | ★ | ★ | 9 |
| Guo 2016 | ★ | ★ | ★ | ★ | ★★ | ★ | ★ | ★ | 9 |
| Fan 2026 | ★ | ★ | ★ | ★ | ★★ | ★ | ★ | ★ | 9 |
| Ciccullo 2026 | ★ | ★ | ★ | ★ | ★★ | ★ | ★ | ★ | 9 |
| Pierone 2025 | ★ | ★ | ★ | ★ | ★★ | ★ | ★ | ★ | 9 |
| Liu 2025 | ★ | ★ | ★ | ★ | ★★ | ★ | ★ | ★ | 9 |
| Lee 2024 | ★ | ★ | ★ | ★ | ★★ | ★ | ★ | ★ | 9 |
| Gan 2024 | ★ | ★ | ★ | ★ | ★★ | ★ | ★ | ★ | 9 |
| Knobel 2023 | ★ | ★ | ★ | ★ | ★★ | ★ | ★ | ★ | 9 |
| Martin-Torres 2026 | ★ | ★ | ★ | ★ | ★★ | ★ | ★ | ★ | 9 |

**Note: Options for each item, one star gives one point:**

① a) truly representative of the average patient (describe) in the community **🟑**; b) somewhat representative of the average patient in the community **🟑**; c) selected group of users eg nurses, volunteers; d) no description of the derivation of the cohort;

② a) drawn from the same community as the exposed cohort **🟑**; b) drawn from a different source; c) no description of the derivation of the non exposed cohort

③ a) secure record (eg surgical records) **🟑**; b) structured interview **🟑**; c) written self report; d) no description

④ a) yes **🟑**; b) no

⑤ a) study controls for patient (select the most important factor) **🟑**; b) study controls for any additional factor **🟑** (This criteria could be modified to indicate specific control for a second important factor.)

⑥ a) independent blind assessment **🟑**; b) record linkage **🟑**; c) self report; d) no description

⑦ a) yes (select an adequate follow up period for outcome of interest) **🟑**; b) no

⑧ a) complete follow up - all subjects accounted for **🟑**; b) subjects lost to follow up unlikely to introduce bias - small number lost - > 20 % (select an adequate %) follow up, or description provided of those lost) **🟑**; c) follow up rate < 20% and no description of those lost; d) no statement

**Supplementary Table S4. Detailed reasons for treatment discontinuation in treatment‑experienced and treatment‑naïve participants receiving BIC/FTC/TAF across included studies**

|  |  |  | **TE** | |  | **TN** | |
| --- | --- | --- | --- | --- | --- | --- | --- |
| **Study** | **Type of drug discontinuation** | **Follow-up period** | **r** | **n** |  | **r** | **n** |
| Yang 2026 | All-cause discontinuation | 24 months | 11 | 268 |  | 1 | 66 |
| Yang 2026 | Discontinuation due to adverse events (DRAEs) | 24 months | 3 | 268 |  | 0 | 66 |
| Yang 2026 | Discontinuation due to lack of effectiveness | 24 months | 2 | 268 |  | 0 | 66 |
| Yang 2026 | Discontinuation due to participant decision | 24 months | 6 | 268 |  | 1 | 6 |
| Inciarte 2025 | All-cause discontinuation | 12 months | 12 | 187 |  | 1 | 62 |
| Inciarte 2025 | Discontinuation due to drug-related adverse events (DRAEs) | 12 months | 5 | 187 |  | 0 | 62 |
| Inciarte 2025 | Discontinuation due to participant decision | 12 months | 3 | 187 |  | 0 | 62 |
| Inciarte 2025 | Discontinuation due to death | 12 months | 1 | 187 |  | 0 | 62 |
| Inciarte 2025 | Discontinuation due to investigator's decision | 12 months | 1 | 187 |  | 1 | 62 |
| Inciarte 2025 | Discontinuation due to lack of efficacy | 12 months | 1 | 187 |  | 0 | 62 |
| Inciarte 2025 | Discontinuation due to missing data | 12 months | 1 | 187 |  | 0 | 62 |
| Trizzino 2025 | All-cause discontinuation | 24 months | 22 | 177 |  | 10 | 37 |
| Trizzino 2025 | Discontinuation due to patient choice | Overall (median 3.4 years) | 10 | 31 |  | 3 | 10 |
| Trizzino 2025 | Discontinuation due to toxicity (adverse events) | Overall (median 3.4 years) | 8 | 31 |  | 1 | 10 |
| Trizzino 2025 | Discontinuation due to simplification | Overall (median 3.4 years) | 7 | 31 |  | 0 | 10 |
| Trizzino 2025 | Discontinuation due to virological failure | Overall (median 3.4 years) | 1 | 31 |  | 1 | 10 |
| Trizzino 2025 | Discontinuation due to drug-drug interactions | Overall (median 3.4 years) | 0 | 31 |  | 2 | 10 |
| Trizzino 2025 | Discontinuation due to cardiovascular prevention | Overall (median 3.4 years) | 4 | 31 |  | 1 | 10 |
| Trizzino 2025 | Discontinuation due to death | Overall (median 3.4 years) | 0 | 31 |  | 2 | 10 |
| Ma 2025 | All-cause discontinuation | 48 weeks | 2 | 143 |  | 4 | 149 |
| Ma 2025 | Discontinuation due to financial reasons | 48 weeks | 0 | 143 |  | 2 | 149 |
| Ma 2025 | Discontinuation due to loss to follow-up / no data | 48 weeks | 2 | 143 |  | 2 | 149 |
| Kim 2025 | All-cause discontinuation | 24 months | 2 | 52 |  | 0 | 36 |
| Kim 2025 | Discontinuation due to participant decision | 24 months | 1 | 52 |  | 0 | 36 |
| Kim 2025 | Discontinuation due to lack of effectiveness | 24 months | 1 | 52 |  | 0 | 36 |
| Kim 2025 | Discontinuation due to drug-related adverse events (DRAEs) | 24 months | 0 | 52 |  | 0 | 36 |
| Cecchini 2025 | All-cause discontinuation | 24 weeks | 8 | 2356 |  | 1 | 315 |
| Cecchini 2025 | All-cause discontinuation | 48 weeks | 9 | 2356 |  | 1 | 315 |
| Cecchini 2025 | Discontinuation due to intolerance | 24 weeks | 1 | 2356 |  | 0 | 315 |
| Cecchini 2025 | Discontinuation due to renal toxicity | 24 weeks | 1 | 2356 |  | 0 | 315 |
| Cecchini 2025 | Discontinuation due to weight gain | 24 + 48 weeks | 3 | 2356 |  | 0 | 315 |
| Cecchini 2025 | Discontinuation due to patient preference | 24 + 48 weeks | 1 | 2356 |  | 0 | 315 |
| Cecchini 2025 | Discontinuation due to hepatic toxicity | 48 weeks | 1 | 2356 |  | 0 | 315 |
| Cecchini 2025 | Discontinuation due to other causes | 24 + 48 weeks | 5 | 2356 |  | 0 | 315 |
| Cecchini 2025 | Discontinuation due to unknown reasons | 48 weeks | 3 | 2356 |  | 0 | 315 |
| Mendoza 2023 | All-cause discontinuation | Overall（24-48 weeks） | 21 | 119 |  | 5 | 12 |
| Mendoza 2023 | Discontinuation due to adverse events | Overall（24-48 weeks） | 5 | 119 |  | 0 | 12 |
| Mendoza 2023 | Discontinuation due to lost to follow-up | Overall（24-48 weeks） | 11 | 119 |  | 1 | 12 |
| Mendoza 2023 | Discontinuation due to death | Overall（24-48 weeks） | 1 | 119 |  | 1 | 12 |
| Mendoza 2023 | Discontinuation due to pregnancy | Overall（24-48 weeks） | 3 | 119 |  | 1 | 12 |
| Mendoza 2023 | Discontinuation due to treatment simplification | Overall（24-48 weeks） | 0 | 119 |  | 1 | 12 |
| Mendoza 2023 | Discontinuation due to drug interactions | Overall（24-48 weeks） | 0 | 119 |  | 1 | 12 |
| Kong 2023 | All-cause discontinuation | 24 weeks | 4 | 177 |  | 2 | 32 |
| Kong 2023 | Discontinuation due to economic difficulties | 24 weeks | 4 | 177 |  | 2 | 32 |
| Kong 2023 | Discontinuation due to transfer to other institution | 24 weeks | 1 | 177 |  | 1 | 32 |
| Kong 2023 | Discontinuation due to adverse events | 24 weeks | 0 | 177 |  | 0 | 32 |
| Monforte 2024 | All-cause discontinuation | Overall (median 69.8 weeks for TN; 146.3 weeks for TE) | 294 | 1655 |  | 166 | 933 |
| Monforte 2024 | Discontinuation due to simplification | Overall (median 69.8 weeks for TN; 146.3 weeks for TE) | 170 | 1655 |  | 69 | 933 |
| Monforte 2024 | Discontinuation due to toxicity/intolerance | Overall (median 69.8 weeks for TN; 146.3 weeks for TE) | 50 | 1655 |  | 32 | 933 |
| Monforte 2024 | Discontinuation due to other causes | Overall (median 69.8 weeks for TN; 146.3 weeks for TE) | 57 | 1655 |  | 52 | 933 |
| Monforte 2024 | Discontinuation due to patient decision | Overall (median 69.8 weeks for TN; 146.3 weeks for TE) | 12 | 1655 |  | 2 | 933 |
| Monforte 2024 | Discontinuation due to virological failure | Overall (median 69.8 weeks for TN; 146.3 weeks for TE) | 5 | 1655 |  | 11 | 933 |
| Nasreddine 2023 | All-cause discontinuation | 48 weeks | 97 | 1593 |  | 34 | 408 |
| Nasreddine 2023 | Discontinuation due to adverse events (any) | 48 weeks | 33 | 1593 |  | 15 | 408 |
| Nasreddine 2023 | Discontinuation due to CNS/psychiatric toxicity | 48 weeks | 5 | 1593 |  | 3 | 408 |
| Nasreddine 2023 | Discontinuation due to gastrointestinal toxicity | 48 weeks | 5 | 1593 |  | 3 | 408 |
| Nasreddine 2023 | Discontinuation due to switch to 2-drug regimen | 48 weeks | 21 | 1593 |  | 5 | 408 |
| Nasreddine 2023 | Discontinuation due to pregnancy | 48 weeks | 5 | 1593 |  | 2 | 408 |
| Yokomaku 2025 | All-cause discontinuation | 12 months | 3 | 84 |  | 4 | 116 |
| Yokomaku 2025 | Discontinuation due to DRAEs | 12 months | 3 | 84 |  | 1 | 116 |
| Yokomaku 2025 | Discontinuation due to investigator's decision | 12 months | 0 | 84 |  | 2 | 116 |
| Yokomaku 2025 | Discontinuation due to participant decision | 12 months | 0 | 84 |  | 1 | 116 |

**Supplementary Table S5. Summary of the specific reasons for discontinuation of BIC/FTC/TAF vs. DTG/3TC**

| **Study** | **Type of drug discontinuation** | **Follow-up period** | **BIC/FTC/TAF** | **DTG/3TC** |
| --- | --- | --- | --- | --- |
| Ghosn 2026 | Discontinuation due to adverse events (AEs) | 48 weeks | 3/255 (1.2%) | 3/254 (1.2%) |
| Ghosn 2026 | Discontinuation due to meeting confirmed virologic withdrawal (CVW) criteria | 48 weeks | 7/255 (3.0%) | 7/255 (3.0%) |
| Guo 2026 | All-cause discontinuation / regimen switch | 48 weeks | 31/402 (7.7%) | 8/97 (8.2%) |
| Perez-Valero 2026 | All-cause discontinuation | 48 weeks | 5/41 (12.2%) | 6/39 (15.4%) |
| Perez-Valero 2026 | Discontinuation due to adverse events (any) | 48 weeks | 4/41 (9.8%) | 3/39 (7.7%) |
| Perez-Valero 2026 | Discontinuation due to NP-AEs | 48 weeks | 3/41 (7.3%) | 2/39 (5.1%) |
| Perez-Valero 2026 | Irritability | 48 weeks | 1/41 (2.4%) | 0/39 (0%) |
| Perez-Valero 2026 | Depressive symptoms | 48 weeks | 1/41 (2.4%) | 1/39 (2.6%) |
| Perez-Valero 2026 | Headache + insomnia | 48 weeks | 1/41 (2.4%) | 0/39 (0%) |
| Perez-Valero 2026 | Anxiety | 48 weeks | 0/41 (0%) | 1/39 (2.6%) |
| Perez-Valero 2026 | Suicidality (investigator-mandated) | 48 weeks | 0/41 (0%) | 1/39 (2.6%)† |
| Perez-Valero 2026 | Discontinuation due to non-NP-AE | 48 weeks | 1/41 (2.4%) | 1/39 (2.6%) |
| Perez-Valero 2026 | Gastrointestinal intolerance | 48 weeks | 1/41 (2.4%) | 0/39 (0%) |
| Perez-Valero 2026 | Tubular dysfunction | 48 weeks | 0/41 (0%) | 1/39 (2.6%) |
| Perez-Valero 2026 | Discontinuation due to loss to follow-up | 48 weeks | 1/41 (2.4%) | 1/39 (2.6%) |
| Perez-Valero 2026 | Discontinuation due to withdrawal of consent (unrelated to AEs) | 48 weeks | 1/41 (2.4%) | 0/39 (0%) |
| Perez-Valero 2026 | Discontinuation due to death (unknown cause) | 48 weeks | 0/41 (0%) | 1/39 (2.6%) |
| Yang 2026b | All-cause discontinuation | 48 weeks | 63/744 (8.5%)* | 36/442 (8.1%) |
| Yang 2026b | Discontinuation due to adverse effects | 48 weeks | 19/744 (2.6%) | 10/442 (2.3%) |
| Yang 2026b | Discontinuation due to loss to follow-up | 48 weeks | 17/744 (2.3%) | 5/442 (1.1%) |
| Yang 2026b | Discontinuation due to death | 48 weeks | 2/744 (0.3%) | 1/442 (0.2%) |
| Yang 2026b | Discontinuation due to transfer | 48 weeks | 7/744 (0.9%) | 8/442 (1.8%) |
| Yang 2026b | Discontinuation due to missing data | 48 weeks | 18/744 (2.4%) | 12/442 (2.7%) |
| Pierone 2025 | All-cause discontinuation | Median 15/16 months | 631/3713 (17.0%) | 443/2327 (19.0%) |
| Pierone 2025 | Treatment-related discontinuations (combined) | Median 15/16 months | 38/3713 (1.0%) | 40/2327 (1.7%) |
| Pierone 2025 | Virologically unsuppressed | Median 15/16 months | 19/3713 (0.5%) | 22/2327 (0.9%) |
| Pierone 2025 | Adverse diagnosis / side effects | Median 15/16 months | 17/3713 (0.5%) | 18/2327 (0.8%) |
| Pierone 2025 | Non-treatment-related discontinuations (combined) | Median 15/16 months | 525/3713 (14.1%) | 359/2327 (15.4%) |
| Pierone 2025 | Switch to long-acting injectable ARVs | Median 15/16 months | 65/3713 (1.8%) | 66/2327 (2.8%) |
| Pierone 2025 | Treatment gap >45 days | Median 15/16 months | 345/3713 (9.3%) | 163/2327 (7.0%) |
| Pierone 2025 | Provider choice | Median 15/16 months | 135/3713 (3.6%) | 140/2327 (6.0%) |
| Pierone 2025 | Patient choice | Median 15/16 months | NR (<5) | NR (<5) |
| Pierone 2025 | Pregnancy | Median 15/16 months | 7/3713 (0.2%) | NR (<5) |
| Pierone 2025 | Access issues | Median 15/16 months | NR (<5) | 0/2327 (0%) |
| Pierone 2025 | Unknown reason for discontinuation | Median 15/16 months | 84/3713 (2.3%) | 68/2327 (2.9%) |
| Pierone 2025 | Discontinuation due to death | Median 15/16 months | 22/3713 (0.6%) | 9/2327 (0.4%) |
| Pierone 2025 | Discontinuation due to loss to follow-up | Median 15/16 months | 104/3713 (2.8%) | 55/2327 (2.4%) |
| Ciccullo 2026 | Discontinuation due to toxicity | 48 weeks | 7/447 (1.6%) | 6/309 (1.9%) |
| Ciccullo 2026 | GI toxicity | 48 weeks | 4/447 (0.9%) | 2/309 (0.6%) |
| Ciccullo 2026 | Weight gain | 48 weeks | 3/447 (0.7%) | 2/309 (0.6%) |
| Ciccullo 2026 | CNS toxicity | 48 weeks | 0/447 (0%) | 1/309 (0.3%) |
| Ciccullo 2026 | Hypersensitivity reaction | 48 weeks | 0/447 (0%) | 1/309 (0.3%) |
| Lee 2024 | All-cause discontinuation | 48 weeks | 40/520 (7.7%) | 24/532 (4.5%) |
| Lee 2024 | Increased weight or obesity | 48 weeks | 8/520 (1.5%) | 2/532 (0.4%) |
| Lee 2024 | Insomnia | 48 weeks | 4/520 (0.8%) | 4/532 (0.8%) |
| Lee 2024 | Nausea/diarrhea | 48 weeks | 4/520 (0.8%) | 1/532 (0.2%) |
| Lee 2024 | Low level viremia | 48 weeks | 4/520 (0.8%) | 1/532 (0.2%) |
| Lee 2024 | Renal function deterioration | 48 weeks | 3/520 (0.6%) | 2/532 (0.4%) |
| Lee 2024 | Hyperlipidemia | 48 weeks | 3/520 (0.6%) | 0/532 (0%) |
| Lee 2024 | Fatigue | 48 weeks | 2/520 (0.4%) | 0/532 (0%) |
| Lee 2024 | Blood sugar poor control | 48 weeks | 2/520 (0.4%) | 0/532 (0%) |
| Lee 2024 | Pruritus | 48 weeks | 2/520 (0.4%) | 5/532 (0.9%) |
| Lee 2024 | Virological failure | 48 weeks | 2/520 (0.4%) | 1/532 (0.2%) |
| Lee 2024 | Headache/dizziness | 48 weeks | 1/520 (0.2%) | 2/532 (0.4%) |
| Lee 2024 | Drug-drug interaction | 48 weeks | 1/520 (0.2%) | 0/532 (0%) |
| Lee 2024 | Completed LTBI treatment, shift back | 48 weeks | 1/520 (0.2%) | 2/532 (0.4%) |
| Lee 2024 | Numbness | 48 weeks | 1/520 (0.2%) | 1/532 (0.2%) |
| Lee 2024 | Anxiety | 48 weeks | 0/520 (0%) | 1/532 (0.2%) |
| Gan 2024 | All-cause discontinuation | 48 weeks | 23/131 (17.6%) | 16/145 (11.0%) |
| Gan 2024 | Lost to follow-up | 48 weeks | 8/131 (6.1%) | 4/145 (2.8%) |
| Gan 2024 | Changed ART regimen (economic reasons) | 48 weeks | 3/131 (2.3%) | 0/145 (0%) |
| Gan 2024 | No 48-week data (transferred hospital) | 48 weeks | 2/131 (1.5%) | 0/145 (0%) |
| Gan 2024 | Stopped ART (non-efficacy/intolerance) | 48 weeks | 1/131 (0.8%) | 0/145 (0%) |
| Gan 2024 | Death | 48 weeks | 1/131 (0.8%) | 1/145 (0.7%) |
| Gan 2024 | Discontinuation due to adverse events | 48 weeks | 0/131 (0%) | 0/145 (0%) |
| Mendoza 2023 | All-cause discontinuation (TE) | Median: 57 weeks | 21/119 (17.6%) | 32/134 (23.9%) |
| Mendoza 2023 | Lost to follow-up (TE) | Median: 57 weeks | 11/119 (9.2%) | 16/134 (11.9%) |
| Mendoza 2023 | Adverse events (TE) | Median: 57 weeks | 6/119 (5.0%) | 5/134 (3.7%) |
| Mendoza 2023 | Death (non-ART-related) (TE) | Median: 57 weeks | 1/119 (0.8%) | 3/134 (2.2%) |
| Mendoza 2023 | Pregnancy (TE) | Median: 57 weeks | 3/119 (2.5%) | 2/134 (1.5%) |
| Mendoza 2023 | Virological failure (TE) | Median: 57 weeks | 0/119 (0%) | 2/134 (1.5%) |
| Mendoza 2023 | Treatment non-compliance (TE) | Median: 57 weeks | 0/119 (0%) | 2/134 (1.5%) |
| Mendoza 2023 | Participation in clinical trial (TE) | Median: 57 weeks | 0/119 (0%) | 2/134 (1.5%) |
| Mendoza 2023 | Treatment simplification (TE) | Median: 57 weeks | 0/119 (0%) | 0/134 (0%) |
| Mendoza 2023 | All-cause discontinuation (TN) | Median: 26 weeks | 5/12 (41.7%) | 5/27 (18.5%) |
| Mendoza 2023 | Lost to follow-up (TN) | Median: 26 weeks | 1/12 (8.3%) | 3/27 (11.1%) |
| Mendoza 2023 | Adverse events (TN) | Median: 26 weeks | 0/12 (0%) | 2/27 (7.4%) |
| Mendoza 2023 | Death (TN) | Median: 26 weeks | 1/12 (8.3%) | 0/27 (0%) |
| Mendoza 2023 | Treatment simplification (TN) | Median: 26 weeks | 1/12 (8.3%) | 0/27 (0%) |
| Mendoza 2023 | Drug-drug interactions (TN) | Median: 26 weeks | 1/12 (8.3%) | 0/27 (0%) |
| Mendoza 2023 | Pregnancy (TN) | Median: 26 weeks | 1/12 (8.3%) | 2/27 (7.4%) |

**Note:**

**Supplementary Table S6. Sensitivity analysis**

| **Question** | **Outcome** | **Study omitted** | **OR (95% CI)** |
| --- | --- | --- | --- |
| TE vs. TN | All-cause discontinuation - 48 weeks/12 months | Cecchini 2025 | 0.80 (0.55, 1.16) |
|  |  | Inciarte 2025 | 0.73 (0.50, 1.07) |
|  |  | Ma 2025 | 0.83 (0.57, 1.21) |
|  |  | Nasreddine 2023 | 1.30 (0.57, 2.96) |
|  |  | YokomakuI 2025 | 0.80 (0.55, 1.16) |
|  |  | **Total (5 studies)** | 0.81 (0.56, 1.17) |
| TE vs. TN | All-cause discontinuation - 24 months | Trizzino 2025 | 2.78 (0.35, 21.94) |
|  |  | Yang 2026 | 0.38 (0.16, 0.90) |
|  |  | **Total (2 studies)** | 0.61 (0.29, 1.29) |
| TE vs. TN | Discontinuation due to adverse events - 48 weeks/12 months | Inciarte 2025 | 0.68 (0.37, 1.23) |
|  |  | Nasreddine 2023 | 4.03 (0.65, 24.97) |
|  |  | YokomakuI 2025 | 0.65 (0.36, 1.18) |
|  |  | **Total (3 studies)** | 0.77 (0.43, 1.37) |
| BIC/FTC/TAF vs DTG/3TC | RCT - Treatment-Experienced - 48 weeks/12 months | Perez-Valero 2026 | 0.49 (0.04, 5.48) |
|  |  | Serrano-Villar 2026 | 1.31 (0.36, 4.70) |
|  |  | **Total (2 studies)** | 1.04 (0.34, 3.16) |
| BIC/FTC/TAF vs DTG/3TC | Cohort - Treatment-Naïve - 48 weeks/12 months | Fan 2026 | 0.99 [0.68, 1.42] |
|  |  | Guo 2026 | 0.96 [0.64, 1.45] |
|  |  | Liu 2025 | 1.01 [0.71, 1.43] |
|  |  | Martin-Torres 2026 | 0.97 [0.68, 1.38] |
|  |  | Sun 2026 | 0.87 [0.56, 1.34] |
|  |  | Yang 2026b | 1.09 [0.75, 1.60] |
|  |  | **Total (6 studies)** | 0.98 [0.70, 1.39] |
| BIC/FTC/TAF vs DTG/3TC | All-cause discontinuation - TN population - 48 weeks | Guo 2026 | 1.04 [0.68, 1.60] |
|  |  | Yang 2026b | 0.93 [0.41, 2.09] |
|  |  | **Total (2 studies)** | 1.02 [0.70, 1.49] |
| BIC/FTC/TAF vs DTG/3TC | Discontinuation due to adverse events - TN population - 48 weeks | Ghosn 2026 | 1.13 [0.52, 2.46] |
|  |  | Yang 2026b | 1.00 [0.20, 4.98] |
|  |  | **Total (2 studies)** | 1.11 [0.55, 2.22] |
